# Characterization of cancer-associated IDH2 mutations that differ in tumorigenicity, chemosensitivity and 2-hydroxyglutarate production

**DOI:** 10.18632/oncotarget.26848

**Published:** 2019-04-12

**Authors:** Kevin P. Kotredes, Roshanak Razmpour, Evan Lutton, Mercedes Alfonso-Prieto, Servio H. Ramirez, Ana M. Gamero

**Affiliations:** ^1^ Department of Medical Genetics and Molecular Biochemistry, Lewis Katz School of Medicine, Temple University, Philadelphia, PA, USA; ^2^ Department of Pathology, Lewis Katz School of Medicine, Temple University, Philadelphia, PA, USA; ^3^ Department of Inorganic and Organic Chemistry, University of Barcelona, Barcelona, Spain; ^4^ Computational Biomedicine, Institute for Advanced Simulation IAS-5 and Institute of Neuroscience and Medicine INM-9, Forschungszentrum Jülich, Jülich, Germany; ^5^ C. and O. Vogt Institute for Brain Research, Medical Faculty, Heinrich-Heine University Düsseldorf, Düsseldorf, Germany

**Keywords:** glioblastoma, IDH2, tumorigenesis, chemotherapy, biomarker

## Abstract

The family of isocitrate dehydrogenase (IDH) enzymes is vital for cellular metabolism, as IDH1 and IDH2 are required for the decarboxylation of isocitrate to α-ketoglutarate. Heterozygous somatic mutations in IDH1 or IDH2 genes have been detected in many cancers. They share the neomorphic production of the oncometabolite (R)-2-hydroxyglutarate [(R)-2-HG]. With respect to IDH2, it is unclear whether all IDH2 mutations display the same or differ in tumorigenic properties and degrees of chemosensitivity. Here, we evaluated the three most frequent IDH2 mutations occurring in cancer. The predicted changes to the enzyme structure introduced by these individual mutations are supported by the observed production of (R)-2-HG. However, their tumorigenic properties, response to chemotherapeutic agents, and baseline activation of STAT3 differed. Paradoxically, the varying levels of endogenous (R)-2-HG produced by each IDH2 mutant inversely correlated with their respective growth rates. Interestingly, while we found that (R)-2-HG stimulated the growth of non-transformed cells, (R)-2-HG also displayed antitumor activity by suppressing the growth of tumors harboring wild type IDH2. The mitogenic effect of (R)-2-HG in immortalized cells could be switched to antiproliferative by transformation with oncogenic RAS. Thus, our findings show that despite their shared (R)-2-HG production, IDH2 mutations are not alike and differ in shaping tumor cell behavior and response to chemotherapeutic agents. Our study also reveals that under certain conditions, (R)-2-HG has antitumor properties.

## INTRODUCTION

Isocitrate dehydrogenase (IDH) enzymes are key components of the tricarboxylic acid (TCA) cycle. NADP^+^-dependent cytoplasmic IDH1 and mitochondrial IDH2, as well as NAD^+^-dependent mitochondrial IDH3, catalyze the oxidative decarboxylation of isocitrate to α-ketoglutarate (α-KG) and produce NADPH or NADH, respectively [1]. Heterozygous somatic mutations in IDH1/2 have been identified in numerous cancers [2, 3], strikingly at high frequency in glioblastoma multiforme [4] and acute myeloid leukemia (AML) [5–7]. High-throughput DNA sequencing of human gliomas identified mutually exclusive somatic mutations in either IDH1 or IDH2 in approximately 70% of secondary glioblastomas and grade II–III gliomas [4]. Cancer-associated IDH mutations have been proposed as early cellular transforming events, arising before p53 alterations or loss of chromosome 1p/19q [8–10]. IDH1 mutations largely occur at arginine (R)132, of which the resulting amino acid substitution can vary (e.g. R132C, R132G, R132H, R132L, or R132S). In contrast, IDH2 mutations are most commonly found at the analogous R172 (e.g. R172K, R172M, R172G, R172S, or R172W) or neighboring R140 (e.g. R140Q, R140L, or R140W).

IDH1/2 mutations confer gain-of-function as they acquire neomorphic enzymatic activity resulting in the production of the oncometabolite (R)-2-hydroxyglutarate [(R)-2-HG; note that (R)-2-HG is also referred to as (D)-2-HG] [11]. Mutated IDH enzymes utilize α-KG, the product of the wild type reaction, as a substrate that is converted to (R)-2-HG. α-KG is known to be an essential co-factor in many enzymatic reactions, including tumor suppressor enzymes, whereas the structurally-related (R)-2-HG can competitively inhibit α-KG-dependent enzymes thus promoting cellular transformation [12]. Increased concentrations of (R)-2-HG or the presence IDH2 mutations have been shown to affect the regulation of chromatin structure [13–15] and not coincidentally correlates with functional disruption of α-KG-dependent DNA-demethylase TET2 [15, 16]. IDH mutations and (R)-2-HG production are also known to antagonize prolyl-hydroxylases, which are regulators of HIF-1α [13, 17], promote cell growth [18], alter cell morphology [19], increase metastatic potential [19], and are associated with overall longer survival in glioma patients [4]. However, while (R)-2-HG production in glioblastoma cells is viewed as a favorable marker for patient survival, it positively correlates with worse prognosis in AML patients, but the cause of this disparity remains unknown [20–22]. Additionally, positive temozolomide (TMZ) chemosensitivity in glioblastoma patients and overall survival were found to be associated with IDH1/2 mutations, but whether this was linked to a specific IDH mutation was not described [23]. It is presumed that all IDH mutations display similar biological activity due to their shared production of (R)-2-HG, however, this premise has not been carefully investigated. In this study, we focused on the characterization of three distinct IDH2 mutations. Each mutant exhibited different biological traits and produced dissimilar concentrations of (R)-2-HG [24, 25]. Most unexpectedly was that while exogenous (R)-2-HG conferred antitumor activity in cell lines that carry wild type IDH2, it enhanced the proliferation of non-transformed cells. Thus, our studies demonstrate that in addition to (R)-2-HG production, knowing the inherent nature of a specific IDH2 somatic mutation may be important in predicting the outcome of response to chemotherapeutic agents.

## RESULTS

We characterized three predominant IDH2 mutants (R172K, R172M, and R140Q) that are often detected in cancer. We first focused our attention on the molecular mechanism involving the change in enzymatic reactivity. Mutations at IDH2-R172 and -R140 (and the homologous IDH1-R132 and -R100) are known to switch-off the WT, or normal, reaction [isocitrate + NADP^+^ → α-KG + CO_2_ + NADPH] while switching-on the mutant, or neomorphic, reaction [α-KG + NADPH → (R)-2-HG + NADP^+^] [11, 25–28]. In particular, we examined how these three IDH2 mutations could affect the catalytically relevant protein conformational states, as well as the binding of substrates with the use of the crystal structure of the IDH2-R140Q mutant in complex with NADPH and an inhibitor (PDB code 4JA8 [29]). We also relied on homology models of IDH2-WT and the IDH2- R172K, -R172M and -R140Q mutants (Supplementary Tables 1 and 2, Supplementary Figure 1) to obtain more information. This structure-based approach has been successfully applied to rationalize biochemical and cell-based data for IDH1 mutants [25, 30, 31] and to understand the relationship between the analogous IDH1-R132H and IDH2-R172K mutations [11].

**Figure 1:**
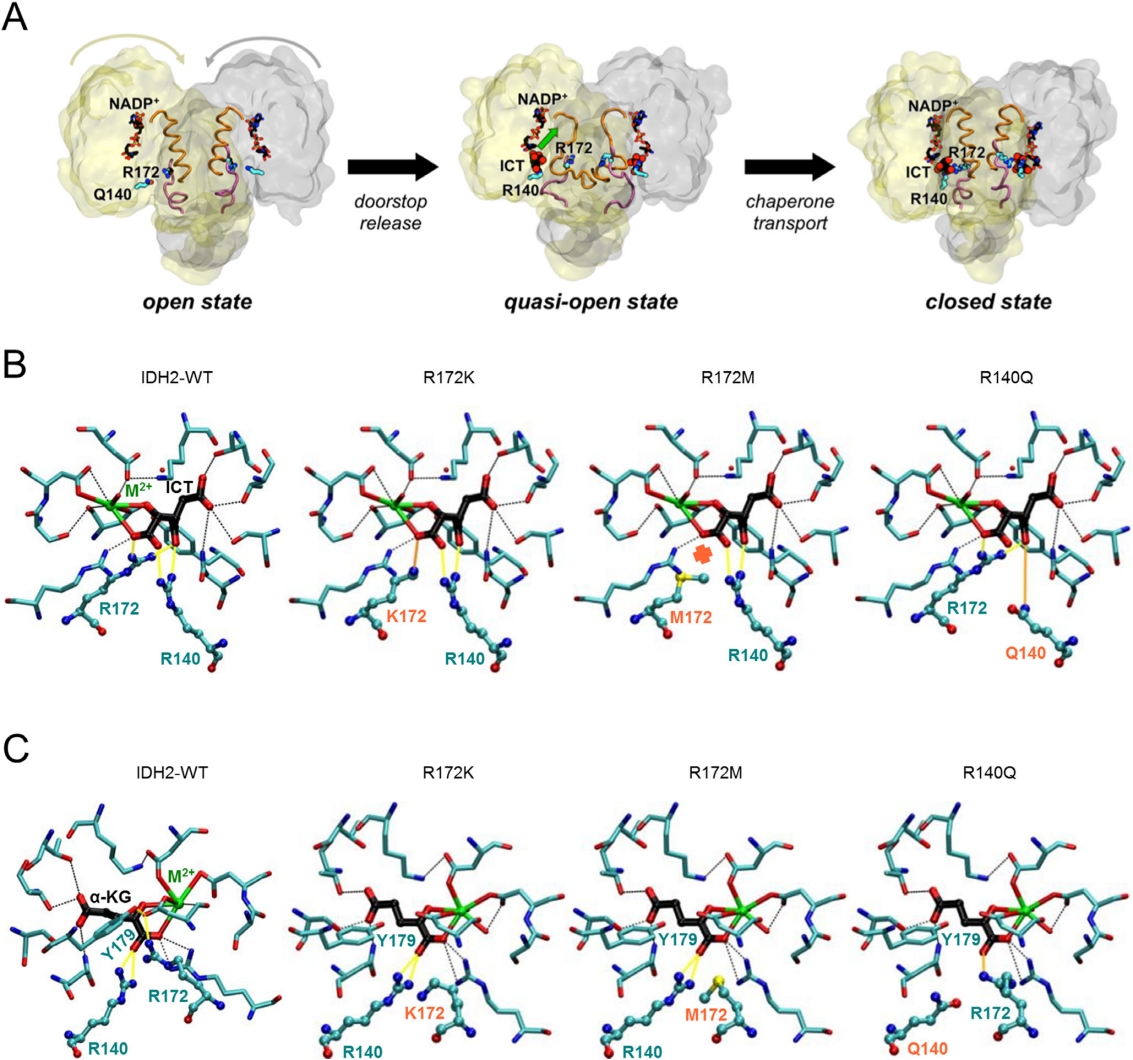
IDH2 mutations at R172 and R140 differentially alter the IDH2 catalytic site structure Schematic of the movement of key residues and co-factors during the normal IDH2 enzymatic process (**A**). Molecular details of the IDH2 catalytic for the wild type enzyme or the three mutants affecting the normal reaction (isocitrate [ICT] bound) (**B**), and the neomorphic reaction (α-ketoglutarate [α-KG] bound) (**C**). Key protein-substrate interactions are highlighted.

First, the possible structural effects of the IDH2-R172 and IDH2-R140 mutations on the enzymatic states involved in the normal reaction were explored. This reaction starts with IDH2 bound to the NADP^+^ cofactor and the enzyme in an open conformation as R172 acts as a “doorstop” by interacting with two aspartate residues (D275 and D279) located at the dimer interface (Figure 1A), similarly to the homologous IDH1-R132 [31]. When the isocitrate substrate binds, it sweeps along R172 allowing the enzyme to adopt a closed conformation and thus properly assembling the active site for the normal reaction to take place. Therefore, R172 mutations are expected to affect the closing of the enzyme, as has also been proposed for the IDH1-R132H mutant [27, 32]. In contrast, R140 is unlikely to alter the conformational transition from the open to the closed state, since it is located farther away from the dimer interface (Figure 1A). Nonetheless, it has been suggested that binding of isocitrate (and thus closing of the enzyme) in IDH1 occurs in two steps [27, 31]: first isocitrate binds to a secondary site on the large domain (quasi-open state in Figure 1A) and then it moves to the catalytic (primary) site, allowing the enzyme to fully close. In this two-step isocitrate binding mechanism, IDH2-R140 acts as a “chaperone,” as does IDH1-R100 [27], guiding the substrate from the secondary to the primary site. Therefore, R140Q is expected to affect isocitrate binding. Once the closed state is reached, isocitrate is bound to the catalytic site, and interacts with both R172 and R140, forming two salt bridges with each residue (Figure 1B, first panel). Therefore, other than the effect on the enzymatic conformational transitions, mutations at R172 and R140 are likely to decrease the affinity of WT-IDH2 for isocitrate, in line with the increased K_m_ measured for the analogous IDH1-R132H and IDH1-R100 mutants [27, 28, 31, 33]. However, each IDH2 mutation is likely to impact the affinity for isocitrate to a different extent, due to the different chemical properties of the mutated residues (Figure 1B, panels 2–4). Based on these findings, we predicted the following ordering from highest to lowest isocitrate K_m_ based on the number of enzyme-substrate interactions: R172M (due to the loss of two salt bridges, compared to WT-IDH2), R140Q (loss of one salt bridge and replacement of the second by a weaker hydrogen bond interaction) and R172K (loss of one salt bridge). Finally, the IDH2-R172K, -R172M and -R140Q mutations are likely to have an additional negative effect on the catalytic efficiency of the enzyme, by analogy with the reduced K_cat_ of the IDH1-R132H, -R132C and -R100A mutants [27, 28, 31]. Altogether, the IDH2-R172 and -R140 mutations would (i) hinder the conformational transitions undergone by the enzyme upon substrate binding, (ii) decrease the affinity for isocitrate in the active site and (iii) impair the catalytic efficiency of the oxidative decarboxylation of isocitrate, and all three effects are aimed at switching-off the normal reaction.

Second, we investigated the possible structural consequences of the IDH2 mutations on the enzymatic states involved in the neomorphic reaction. In the open state (IDH2 in complex with NADPH), mutations at R172 may affect the flexibility of the dimer interface (as for IDH2 bound to NADP^+^, see above), but this effect might be compensated by the higher affinity for NADPH of the mutant enzyme compared to WT-NADP^+^, as proposed for IDH1-R132H [31, 33]. In the quasi-open state (IDH2 in complex with NADPH and α-KG bound in a secondary site), it is unlikely that the R140 “chaperone” residue is necessary to bind α-KG, as it is for isocitrate in the wild type reaction, because α-KG lacks the β-carboxylate group that would interact with R140 (Figure 1A). Finally, in the closed state (IDH2 in complex with NADPH and α-KG bound in the catalytic site), both R172 and R140 mutations would facilitate the neomorphic reaction, but to a different extent. On one hand, the active site of IDH2-WT has a total charge of +3 which is optimized to bind isocitrate (−3) rather than α-KG (−2); therefore, the IDH2-R172M and -R140Q mutations, which remove a positive charge, may allow α-KG to replace isocitrate as the preferred substrate, in line with the analogous IDH1-R132H mutation reducing the competition between isocitrate and α-KG [31]. In contrast, the IDH2-R172K mutant, which keeps the positive charge, would discriminate less between isocitrate and α-KG. On the other hand, the crystal structures of the IDH1-R132 mutant with α-KG bound show that the key catalytic residue Y139 (homologous to IDH2-Y179) is rotated and positioned closer to the substrate compared to WT-IDH1 with isocitrate bound, and this has been proposed to promote direct hydride transfer and conversion to (R)-2-HG [27, 28, 33]. Among the three IDH2 mutants, the R172M mutation would likely favor the most the Y179 conformational change (Figure 1C, compare panels 1 and 3), due to its smaller size and lack of positive charge, and thus would produce the most (R)-2-HG. The R172K mutant would correspond to an intermediate case, even though the lysine residue still has a positive charge, its side-chain is smaller and more flexible than WT arginine, and thus the Y179 rotation would be only partially hindered (Figure 1C, panel 2). In the R140Q mutant, because the WT interaction network R172-D275-Y179 is still present (Figure 1C, panel 4), Y179 would not be able to rotate and adopt the conformation necessary to facilitate the neomorphic reaction. Altogether, the IDH2-R172 and IDH2-R140 mutations would (i) hinder the conformational transitions undergone by the enzyme upon substrate binding, (ii) strengthen the preference to bind α-KG over isocitrate, and (iii) increase the catalytic efficiency of (R)-2-HG production. All three effects favor the switch-on of the neomorphic reaction. Moreover, the structural analysis predicts that the R172M mutation would be most favorable for (R)-2-HG production, followed by R172K and R140Q, provided that the Y179 conformational change is the limiting factor for the conversion of α-KG into (R)-2-HG.

**Figure 2:**
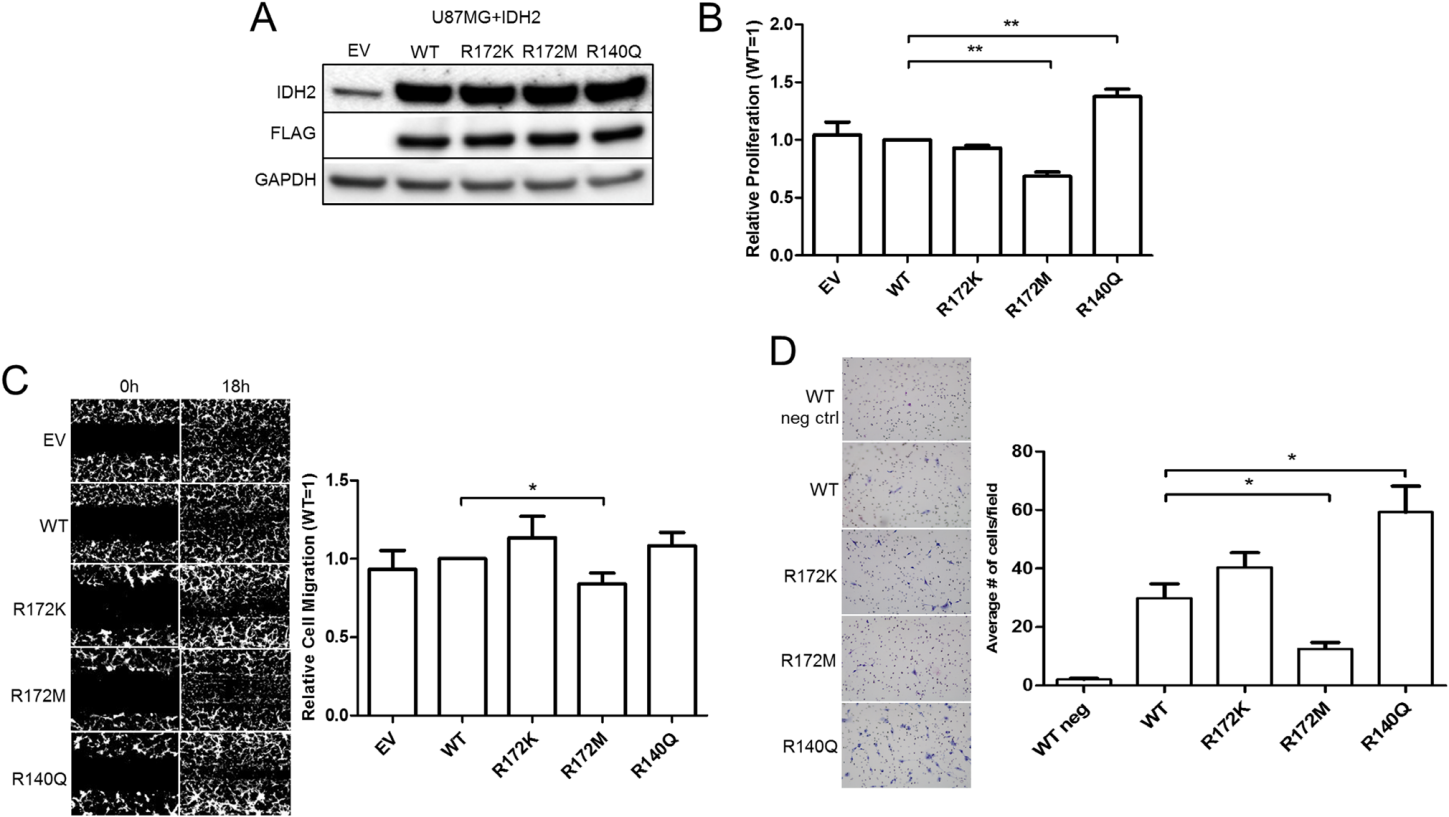
IDH2 mutations alter the tumorigenic properties of U87MG glioblastoma cells Endogenous IDH2 expression in parental U87MG cells expressing empty vector (EV), or overexpression of either wild type (WT) or individual clinically relevant IDH2 mutations (R172K, R172M, and R140Q), confirmed by Western blot analysis (**A**). *In vitro* proliferation assessed by cell counts over 96 h and normalized to WT values (**B**). Assessment of cell migration via wound healing assay by comparing differences in cell density after 18 h, relative to WT values (**C**). Cell invasion of the U87MG+IDH2 panel over 24 h with 10% FBS as a chemoattractant (**D**). Shown are representative images taken at 4X magnification from three independent experiments. ^*^p ≤ 0.05; ^**^p ≤ 0.01.

We proceeded to evaluate the biological significance of the three IDH2 somatic mutations by employing the human UM87G and U251 glioblastoma cell lines; both lines carry wild type (WT) IDH2. Cells were transduced with lentiviral vectors to generate a panel of cell lines to express FLAG-tagged WT, R172M, R172K, or R140Q IDH2 variants (Figure 2A, Supplementary Figure 2), mimicking heterozygous IDH2 found in patients. Immunocytochemistry and confocal microscopy confirmed expression and mitochondrial localization of exogenous IDH2 amongst the pooled populations of the U87MG cell panel (Supplementary Figure 2). We assessed growth rates for each IDH2 mutant and compared them against IDH2-WT. U87MG cells expressing IDH2-R140Q proliferated faster while cells expressing IDH2-R172M cells proliferated slower relative to IDH2-WT cells. No significant differences in growth rates were found between IDH2-WT and IDH2-R172K expressing U87MG cells (Figure 2B) and U251 cells (Supplementary Figure 3B). We also assessed changes in tumor cell migration via scratch assay. We found that IDH2-R172M cells migrated the slowest while IDH2-R172K cells had a tendency to migrate faster in comparison to IDH2-WT (Figure 2C). It is worth noting that IDH2-R140Q also enhanced migration, but this effect was only observed in U251 cells (Supplementary Figure 3C). Another hallmark of tumor cell aggressiveness is invasion through an extracellular matrix. We found IDH2-R172M reduced the capacity of cells to invade. In contrast, IDH2-R140Q enhanced invasion relative to IDH2-WT (Figure 2D).

**Figure 3:**
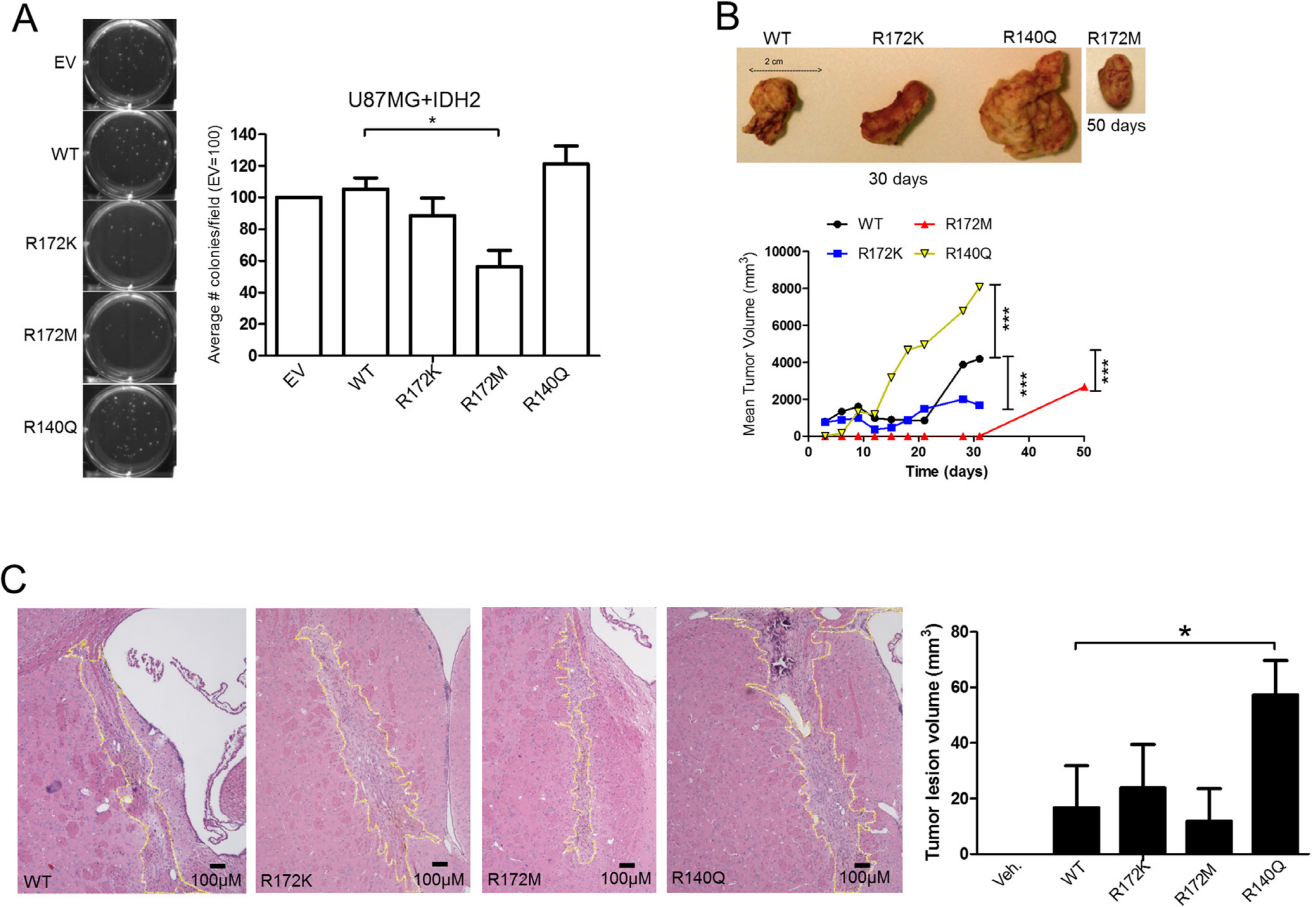
IDH2-R172M and IDH2-R140Q affect tumor growth Soft-agar tumor colony formation of U87MG+IDH2 cell panel assessed at 28 days (**A**). Subcutaneous tumor xenografts of U87MG cells expressing IDH2-WT, IDH2-R172K, IDH2-R172M, or IDH2-R140Q in SCID mice (*n* = 3). Tumor volumes were measured over the course of 50 days (**B**). Orthotopic U87MG tumors in *Rag1*^−/−^ mice (*n* = 3). Brains were collected after 28 days and tumor volumes determined by using 5 μM serial tissue sections (**C**). Shown are representative images taken at 4X magnification. ^*^p ≤ 0.05.

We next measured anchorage-independent growth amongst the IDH2 mutant panel. Consistent with the patterns observed above, IDH2-R172M reduced the number of tumor colonies by more than 40% compared to IDH2-WT. Although IDH2-R140Q increased tumor colony formation, this effect was found not to be statistically significant. IDH2-R172K showed no obvious difference when compared to IDH2-WT (Figure 3A). To translate these findings *in vivo*, our panel of U87MG cells was tested in a tumor xenograft model by transplanting cells subcutaneously into *Rag1*^−/−^ mice. IDH2-R140Q tumors grew more rapidly when compared to IDH2-WT and IDH2-R172K (Figure 3B). In contrast, IDH2-R172M tumors developed at a slower rate, requiring 50 days to detect a palpable tumor. Similar findings were obtained when we employed an orthotopic intracranial tumor model (Figure 3C). Both results confirmed differential *in vivo* growth of glioblastoma cells harboring contrasting IDH2 mutations.

**Figure 4:**
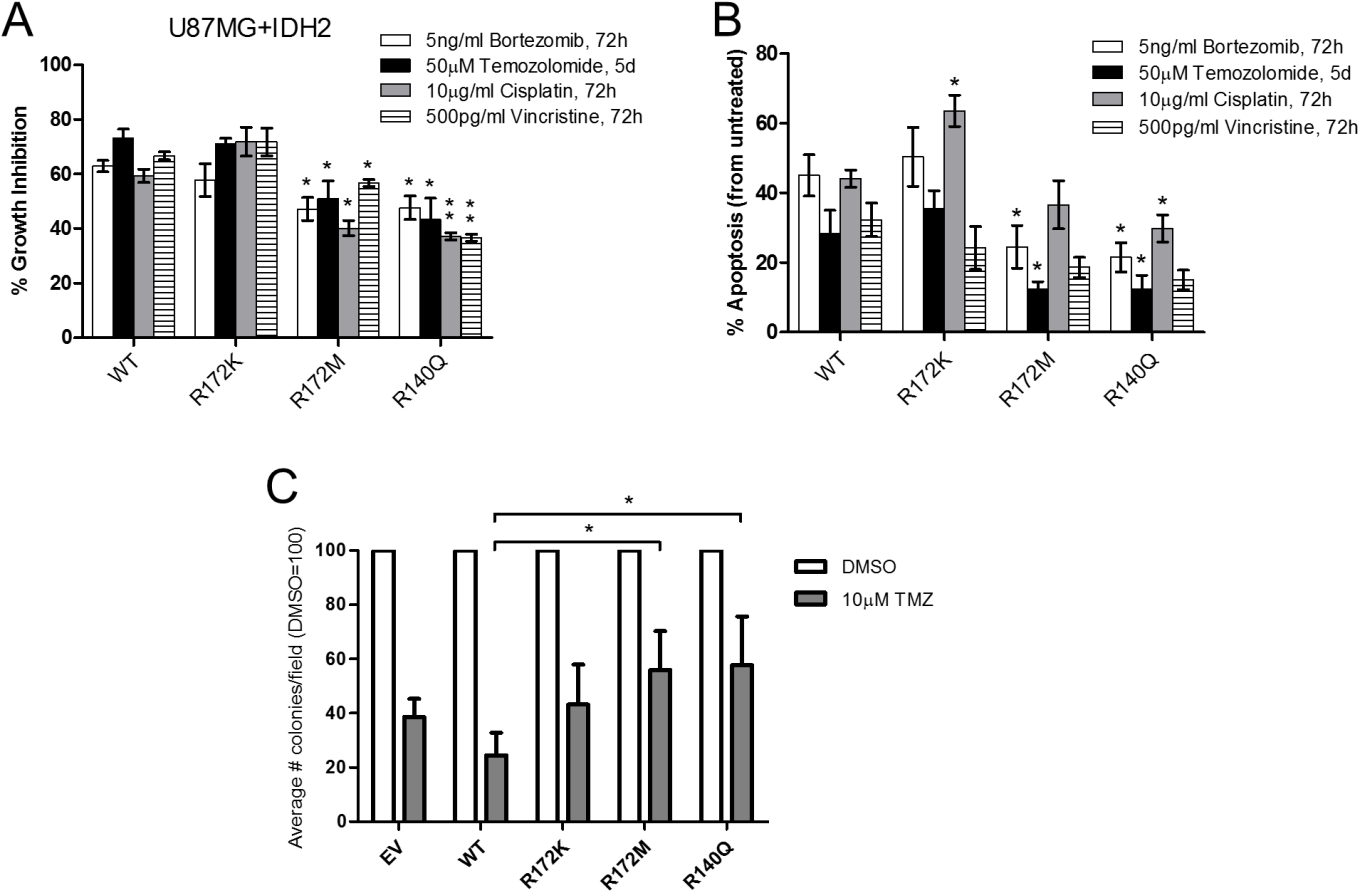
Glioblastoma cells expressing IDH2-R172M and IDH2-R140Q are less responsive to chemotherapeutic drugs Percent growth inhibition determined by cell counts (**A**) and induction of apoptosis by Annexin-V staining (**B**) in response to four different chemotherapeutics in the U87MG+IDH2 cell panels (percent inhibition and apoptosis relative to untreated controls). Effect of temozolomide (TMZ) on tumor colony formation of U87MG+IDH2 cell panel relative to vehicle-treated control (DMSO) over 28 days (**C**). Data presented are from three independent experiments and shown as mean ± SEM. ^*^p ≤ 0.05; ^**^p ≤ 0.01.

We next interrogated whether mutations in IDH2 could affect the response to chemotherapeutic drugs widely used in the standard of care for glioblastoma. The panel of U87MG expressing mutant IDH2 or IDH2-WT was treated with temozolomide, bortezomib, cisplatin, or vincristine and evaluated for changes in cell proliferation and apoptosis. Compared to IDH2-WT, IDH2-R172M and IDH2-R140Q reduced the antitumor effects of all four drugs tested in the range of 20–50% (Figure 4A–4B). In contrast, the response of cells overexpressing IDH2-R172K was comparable to that of IDH2-WT, except for cisplatin, which increased the level of apoptotic cells. These findings were confirmed by soft-agar colony formation assay in which treatment of IDH2-R172M and IDH2-R140Q with temozolomide increased the number of tumor colonies compared to vehicle control-treated cells (Figure 4C).

**Figure 5:**
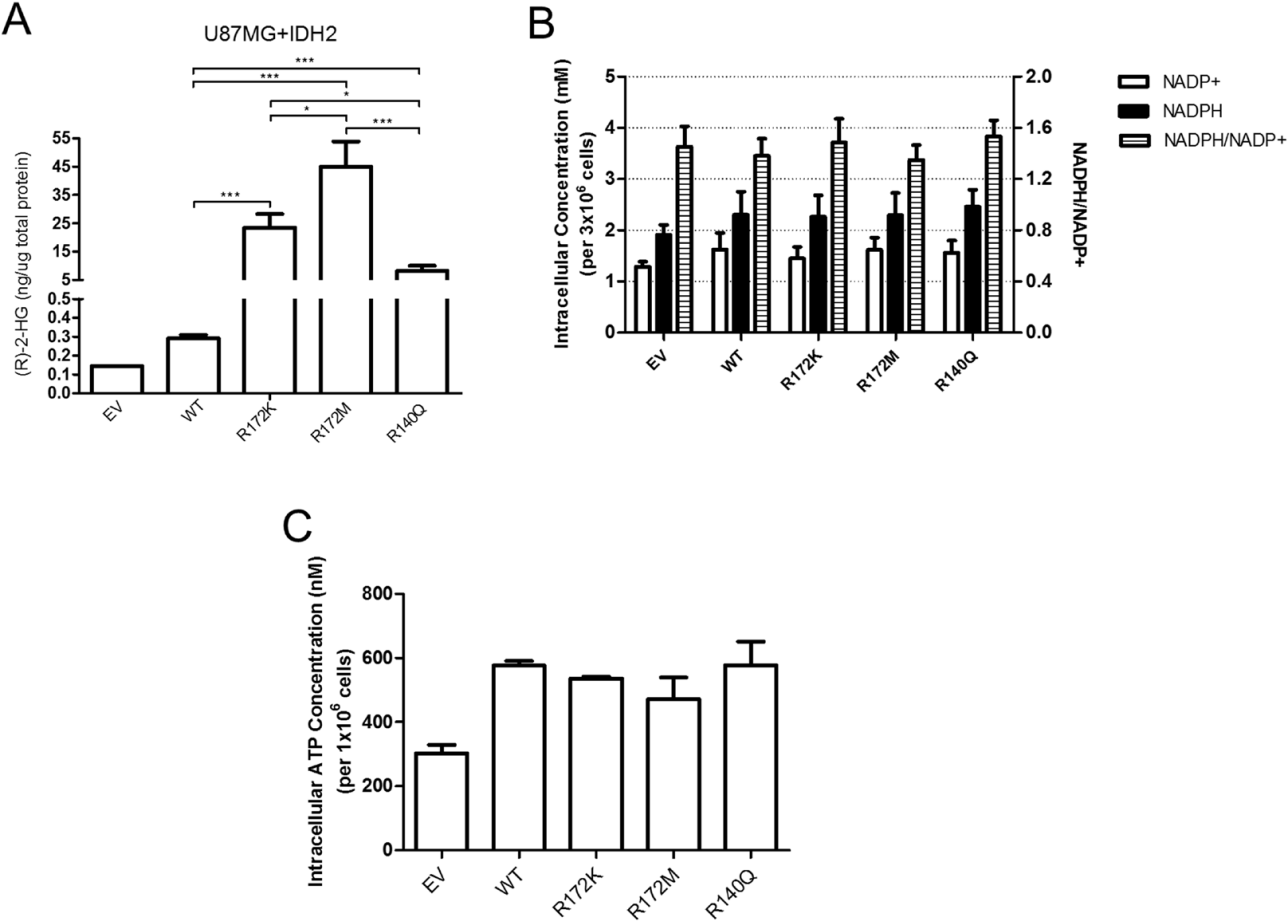
The amount of (R)-2-HG produced in U87MG cells is determined by the nature of the IDH2 mutation while levels of NADP and ATP remain unchanged Intracellular measurement of (R)-2-HG levels in the U87MG+IDH2 panel by gas chromatography-mass spectrometry (GC-MS) (**A**). Data shown are from three independent samples. Levels of NADP^+^, NADPH, NADPH/NADP^+^ ratios (**B**) and ATP (**C**) in U87MG+IDH2 glioblastoma cells expressing IDH2 variants. Data shown are from 3–5 independent samples. ^*^p ≤ 0.05; ^**^p ≤ 0.01; ^***^p ≤ 0.001.

To help explain the phenotypic differences seen with the three IDH2 mutants, we measured intracellular levels of (R)-2-HG. The oncometabolite levels varied significantly amongst the three IDH2 mutations and were inversely correlated with cell growth rates. As expected, over-expression of IDH2-WT caused detectable, but minuscule amounts of (R)-2-HG (Figure 5A) that matched levels found in human patients [28, 34, 35] and cell lines expressing both exogenous and endogenous IDH1/2 transcripts [36]. U87MG cells expressing IDH2-R172M produced the highest amount of (R)-2-HG while IDH2-R140Q cells produced the least when compared to IDH2-R172K cells (Figure 5A). It was shown previously that IDH2-WT protein encoded on one allele may have differential binding affinity to IDH2 mutant proteins encoded on the second allele [24], which might explain differences in the production of (R)-2-HG. However, co-immunoprecipitation of IDH2-WT with individual IDH2-mutants revealed undisturbed heterodimer formation (Supplementary Figure 4).

Intracellular levels of enzymatic cofactors NADP^+^ and NADPH were also measured but no differences between IDH2-WT and the three IDH2 mutants were found (Figure 5B). Similarly, no differences in ATP levels were observed (Figure 5C). Changes in the intracellular concentration of (R)-2-HG can affect cellular behavior by deregulating proper genome epigenetic configuration [12]. However, in our study, no obvious global changes in histone methylation for active (H3K4me3) or repressed (H3K27Me3) transcription were seen when evaluated by immunoblotting (data not shown).

**Figure 6:**
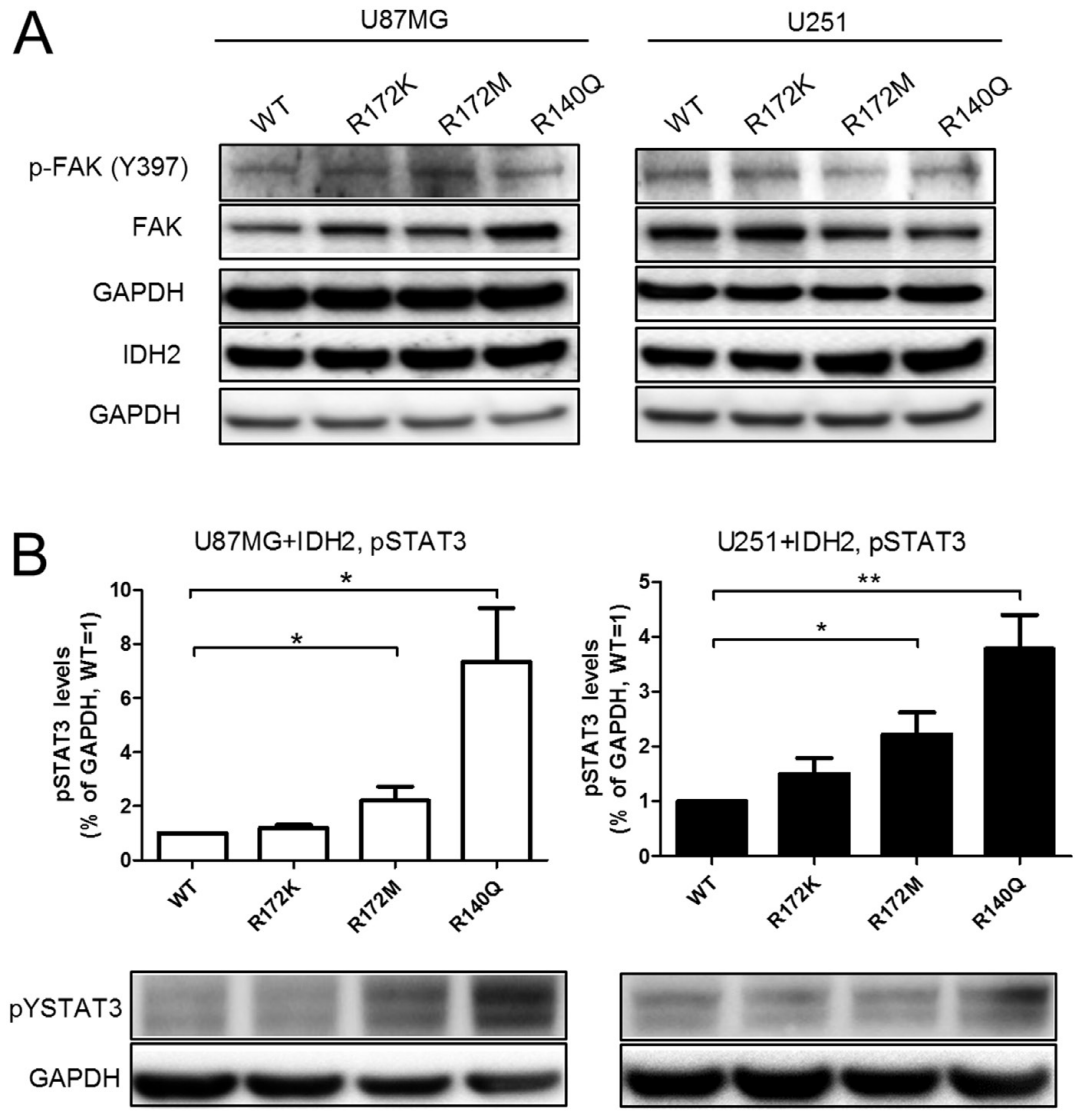
IDH2 mutational status influences baseline activation of STAT3 Phosphorylation status of Y397-FAK and Y705-STAT3 (**A–B**, respectively) in U87MG+IDH2 and U251+IDH2 cell panels were evaluated by western blot analysis. Membranes were re-probed for total FAK and IDH2 expression. GAPDH served as internal loading control. Shown is a representative experiment of three that were performed. Activated STAT3 levels were quantified and normalized to GAPDH expression and relative to IDH2-WT levels set equal to 1. Data shown is a representative experiment of three that were performed. ^*^p ≤ 0.05, ^**^p ≤ 0.01.

At the intracellular signaling level, activation of focal adhesion kinase (FAK), a well-known mediator of cellular motility, was evaluated by western blot analysis. We found no marked differences in the level of activation or protein expression levels of FAK (Figure 6A). Similarly, the baseline activation of stress pathways AKT, ERK, and NF-κB, which are important for cell survival and tumorigenesis, remained unchanged by the expression of mutant IDH2 (data not shown). In contrast, elevated active STAT3 was detected consistently in both U87MG and U251 cells that carry either IDH2-R140Q or IDH2-R172M (Figure 6B). This finding adds support to the role of STAT3 in the tumorigenic features of IDH2 mutations.

**Figure 7:**
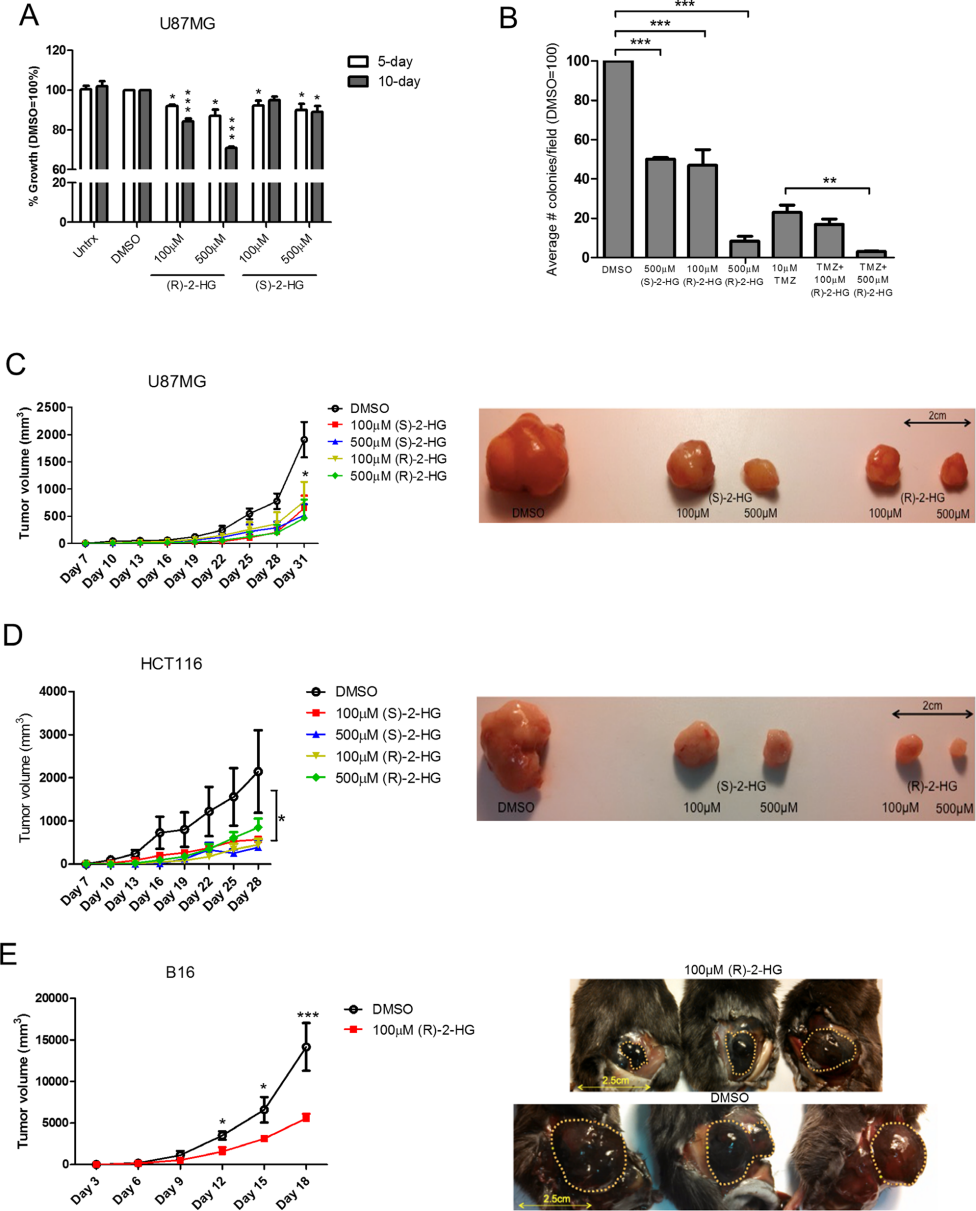
(R)-2-HG inhibits cell proliferation and suppresses tumor growth Parental U87MG cells were treated with vehicle control (DMSO), 100 μM or 500 μM of exogenous (R)-2-HG or (S)-2-HG for 5 and 10 days. Total cell number was determined and normalized to vehicle control (**A**). Data are shown as mean ± SEM of three independent experiments. Tumor colony formation under treatment with (R)-2-HG, TMZ (10 μM), or in combination was determined by soft-agar assay at 28 days (**B**). Data are shown as total colony counts normalized to DMSO control. *Rag1*^−/−^ mice (*n* = 5) with established subcutaneous U87MG (**C**) or HCT116 tumors (**D**) were treated intratumorally every three days with vehicle control (DMSO) vehicle, low (100 μM), or high (500 μM) dose (S)-2-HG or (R)-2-HG over 31 days. Wild type mice with established B16 melanoma tumors were treated with (R)-2-HG only (**E**). ^*^p ≤ 0.05; ^**^p ≤ 0.01; ^***^p ≤ 0.001.

Thus far our data show that IDH2 mutations confer different tumorigenic phenotypes and the levels of (R)-2-HG produced is dictated by the structural nature of the IDH2 mutation (Summarized in Supplementary Table 3). Previous studies report that (R)-2-HG production caused by IDH mutations drives tumorigenesis [37], indicating that (R)-2-HG is an oncometabolite and mitogen [11]. Paradoxically, glioblastoma patients whose tumors harbor mutated IDH1/2 respond better to temozolomide. Therefore, to mirror this condition *in vitro*, we first tested the effect of exogenous (R)-2-HG using parental UM87G cells, which express IDH2-WT. We also tested (S)-2-HG [also known as (L)-2-HG] which is the enantiomer of (R)-2-HG. (S)-2-HG is also produced from α-KG, as a result of the “off-target” activity of malate dehydrogenase (MDH) [38] or loss of L-2-hydroxyglutarate dehydrogenase (L2HGDH) expression [39, 40]. (S)-2-HG is regarded as an oncometabolite as patients with elevated (S)-2-HG have a propensity to develop tumors [39, 41]. We found that (R)-2-HG inhibited the proliferation of U87MG cells while (S)-2-HG had minimal effect (Figure 7A). We then assessed the effect of (R)-2-HG and (S)-2-HG on tumor colony formation. We found that both metabolites inhibited the formation of tumor colonies with (R)-2-HG being more potent than (S)-2-HG, as 500 μM of (S)-2-HG was needed to induce the same effect of 100 μM (R)-2-HG. Furthermore, the addition of (R)-2-HG to temozolomide significantly reduced tumor colony formation (Figure 7B).

**Figure 8:**
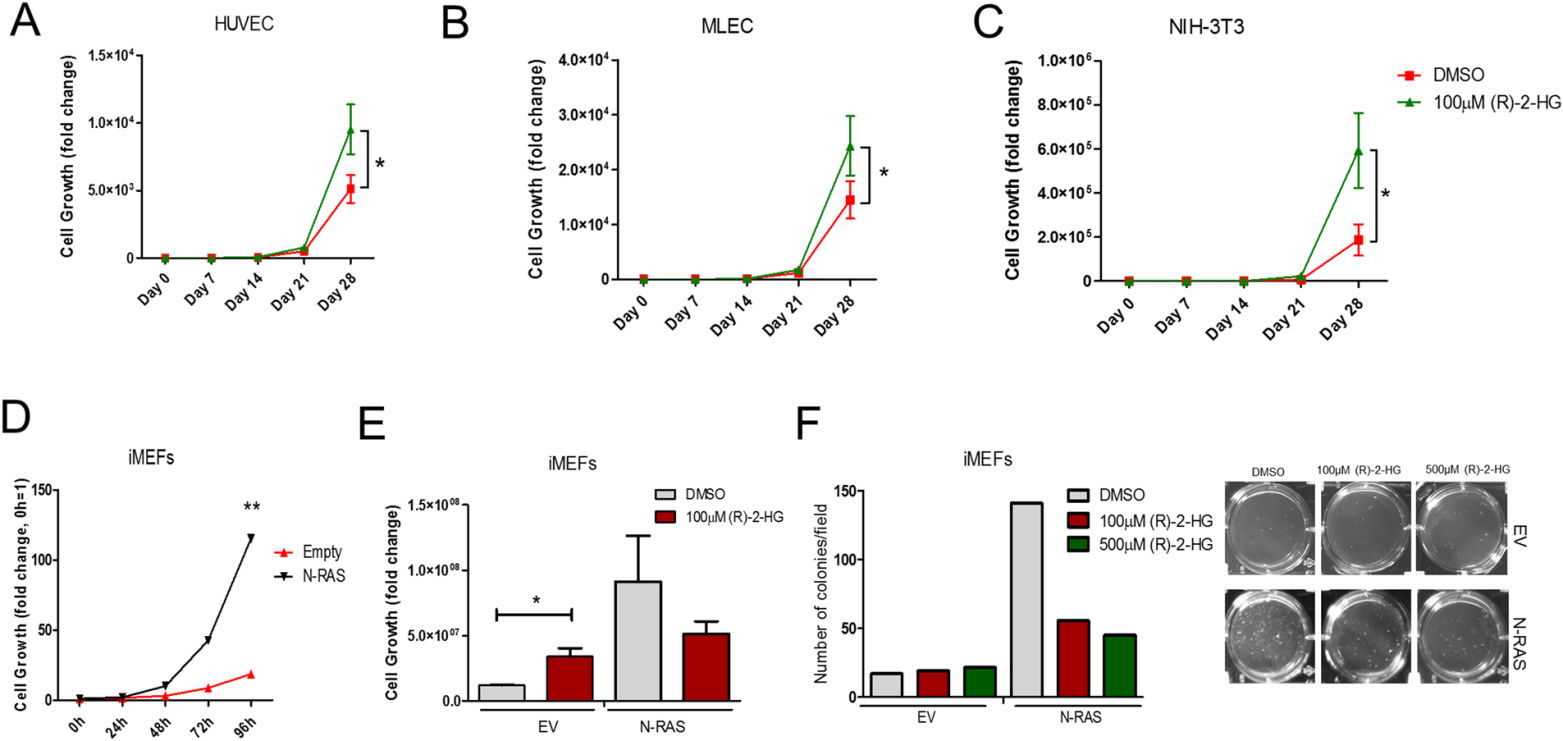
(R)-2-HG suppresses tumor colony formation following transformation with oncogenic Ras Proliferation of HUVEC, MLEC, and NIH-3T3 treated with vehicle (DMSO) or exogenous (R)-2-HG (**A**–**C**). Cell counts were performed over course of 28 days and normalized to vehicle control. (*n* = 3) ^*^p ≤ 0.05. Proliferation of immortalized mouse embryonic fibroblasts (iMEFs) expressing empty vector (EV) or N-RAS was determined by cell counts over 96 h (**D**) and with and without (R)-2-HG treatment after 28 days (**E**). Cell colony formation of iMEFs expressing EV or N-RAS treated with DMSO or (R)-2-HG was determined at 28 days (**F**). (*n* = 2) ^*^p ≤ 0.05.

To confirm the antitumor effects of (R)-2-HG and (S)-2-HG *in vivo*, mice implanted with subcutaneous tumors that are wild type for IDH2 (U87MG, human colorectal HCT116 and murine B16 melanoma) received multiple treatments of each enantiomer. Both (S)-2-HG and (R)-2-HG were equally effective in suppressing tumor growth (Figure 7C–7E). This unexpected finding reveals that (R)-2-HG and (S)-2-HG exert antitumor activity in tumor cells with intact IDH2 function. Other groups have shown that (R)-2-HG is mitogenic, for example, when tested in cytokine-dependent TF-1 cells [18]. We obtained comparable results in which (R)-2-HG enhanced the proliferation of immortalized cells of mouse and human origin (Figure 8A–8C). This raised the question as to what will trigger the antitumor activity of (R)-2-HG. One principal difference between tumor cells and non-transformed cells is the action of an oncogene. To evaluate this possibility, we first transformed immortalized mouse embryonic fibroblasts (iMEFs) with oncogenic N-RAS. As expected, when compared to empty vector control, N-RAS-transformed cells proliferated faster (Figure 8D). We then tested the effect of (R)-2-HG on both cell populations. Treatment of iMEFs with (R)-2-HG increased cell proliferation. N-RAS-transformed iMEFs, in contrast, showed reduced proliferation in response to (R)-2-HG, but this effect did not reach statistical significance. We also tested the effect of (R)-2-HG by soft agar assay and found that iMEFs were not able to form tumor colonies effectively under these conditions. On average, we detected 5–10 colonies for which we were not able to reach any conclusions. However, treatment of N-RAS-transformed iMEFs with (R)-2-HG inhibited tumor colony formation by more than 50% (Figure 8E). This observation demonstrates that oncogenes play a role in switching the mitogenic effect of (R)-2-HG to one that is growth inhibitory.

## DISCUSSION

Otto Warburg first postulated that most cancer cells rely on energy produced predominantly by high rate glycolysis, providing cancer cells with a growth advantage by supplying needed metabolites rather than by oxidation of pyruvate in the mitochondria, like most normal cells [42]. Therefore, alterations to intermediaries of the metabolic pathways, like IDH in the TCA cycle, may be accountable for easing this metabolic shift. Despite a number of metabolic perturbations found in cancer cells, IDH mutations are unique as driver mutations, acting as proto-oncogenes for tumor maintenance [37] and similarly producing a cell state permissive of transformation by alterations to the cell methylome [43]. As hypothesized, we found that IDH2 mutations greatly affected the growth of glioblastoma tumor cells both *in vitro* (Figure 2, Supplementary Figure 3) and *in vivo* (Figure 3). More so, when examined carefully, individual IDH2 mutations displayed differential effects on tumor cell growth. These differences appeared to be directed by the alterations induced by mutations to the catalytic core of the enzyme. Thus, the production of (R)-2-HG is predicted (Figure 1) and substantiated (Figure 5) to differ in cells harboring these mutations [28, 35, 36, 44]. How intracellular (R)-2-HG concentrations influence cell behavior is still a burgeoning focus, nonetheless, many cellular processes have been shown to be affected. Epigenetic changes are suspected to be primary drivers of oncogenic evolution and result in a block in cell differentiation and promotion of cell proliferation in tumor cells [45]. The heterozygosity of all known IDH1/2 mutations paired with the understanding that these enzymes function as dimers suggest that tumor cells remain dependent on a wild type allele of IDH to catalyze the standard reaction found within the TCA cycle [36, 46]. α-KG- (or 2-oxoglutarate-) dependent oxygenases, a family of enzymes involved in methylation of histones and DNA, are greatly affected by both (R)- and (S)-2-HG metabolites [4, 13, 15, 43, 47]. Although we did not detect global differences in the levels of tri-methylated histone H3 lysine residues, indicative of changes in global gene transcription, this does not rule out the possibility of local changes in the epigenetic signature of individual gene promoters. Expression of the genes under the control of these promoters is essential in dictating the behavior of the cell. Due to the numerous findings showing the effects of 2-HG metabolites on epigenetic regulation, it can be safely expected that this ultimately changes the transcriptome and overall cellular make-up. Whether or not there exist specific, coordinated events that drive tumorigenesis or if a general state of cellular stress forces a cell to become cancerous remains undetermined. In addition to differences in tumorigenicity, we found that cellular response to chemotherapeutic agents was affected by IDH2 status. Clinically, positive temozolomide (TMZ) chemosensitivity in glioblastoma patients and overall survival have been shown to be associated with mutated IDH [23]. Our study presents evidence of the importance of knowing the identity of the specific IDH2 mutation (Figure 3). Recent studies indicate that (R)-2-HG can be detected via magnetic resonance imaging (MRI)-based spectroscopy of the brain [48] and in the serum of leukemia and cholangiocarcinoma patients with IDH mutant tumors [49, 50] revealing promising new avenues for cancer detection, staging, and treatment. Perhaps most fascinating is our observation that ‘oncometabolite’ (R)-2-HG is not mitogenic for tumor cells that carry normal IDH2 (Figure 7, Supplementary Figure 5). Quite the opposite, we found that (R)-2-HG reduced tumor colony formation and tumor growth. However, (R)-2-HG does promote *in vitro* growth of non-transformed cells (Figure 8). This has significant implications for understanding how tumors interact with the stroma and indicate (R)-2-HG may have more influence as a paracrine factor than autocrine.

Recently, hybrid QM/MM (quantum mechanics/molecular mechanics) simulations [51] have validated the catalytic mechanism of the normal IDH reaction previously proposed by Aktas and Cook [52]. They also confirmed the crucial role of IDH1 Asp275 and Tyr139 (equivalent to IDH2 Asp314 and Tyr179) in catalysis [51]. This is in line with our observations that the change in position of IDH2-Tyr179 in the mutants is a crucial event to switch the reactivity of IDH from normal to neomorphic (Supplementary Table 1). In addition, biochemical characterization of three different mutants of IDH1 (R132H, R132C, and R132G) [53] show that the IDH1 mutants indeed have a differential production of (R)-2-HG, similar to our results with the IDH2 mutants. Therefore, this would further support our assumptions that one can extrapolate the structural and biochemical data on the IDH1 mutants (IDH1-R132H and -R132C) to the IDH2 mutants (IDH2-R172K and -R172M). On one hand, IDH1-R132H and IDH2-R172K are comparable because, in both cases, the mutated residue (either His or Lys) still carries a positive charge and thus it may still interact with isocitrate. On the other, IDH1-R132C and IDH2-R172M are comparable because, in both cases, the mutation (to either Cys or Met) removes a positive charge and decreases the size of the amino acid at that position, favoring the conformational change of the catalytic Tyr and thus the neomorphic reaction. Here we present a structural comparison of WT and mutant IDH2 in the different states relevant for the normal and neomorphic reactions that offer insight into the molecular basis of the different intracellular (R)-2-HG levels found in our U87MG+IDH2 cell panels (Figure 5). Further confirmation of the hypotheses put forward here warrants an integrative approach, combining X-ray crystallography (to solve the crystal structures of WT and mutant IDH2 in complex with isocitrate and α-KG) and biochemical and kinetic experiments (to determine the affinity of the two substrates and the catalytic activity of WT and mutant IDH2 for both the normal and the neomorphic reaction).

Clinical trials have begun after preclinical studies demonstrated the efficacy of small molecule inhibitors against mutant forms of IDH1 and IDH2 accompanied by a reduction of (R)-2-HG levels, which resulted in a block of de-differentiation and growth suppression of AML tumor cells [29, 54]. These results bring into question the use of IDH2 inhibitor AG-221 [55], which has been shown to be effective in producing durable responses in AML patients. Though AML and gliomas are similar in that they harbor IDH mutations, in glioblastoma cells it is a favorable marker for patient survival while in AML it is correlated with worse prognosis. Therefore, careful consideration must be given to the role of IDH mutations within the progression and maintenance of each of these diseases: the nature of the IDH mutation, the effects of (R)-2-HG concentrations, and the stage of disease when IDH inhibitors are administered. If in fact (R)-2-HG is inhibiting tumor growth, as reported in this study and by another group [56], inhibition of mutant IDH2 enzymes may in actuality promote cancer progression. This raises important questions regarding the function of IDH2 mutations during tumorigenesis as a whole. As a driver mutation, production of (R)-2-HG may cause epigenetic changes leading to chromosomal instability and potentiating further tumorigenic alterations. Nonetheless, after functioning as an oncogene, via (R)-2-HG production, it may switch to tumor suppressor function after initiation of the glial malignancy. If so, AG-221 should not be administered to glioblastoma patients. Furthermore, this also hints at the possibility that mutant IDH2 enzymes may have other effectors that play a role in tumorigenesis, aside from (R)-2-HG. Or perhaps the effects of the oncometabolite are far more extensive, affecting more than the currently appreciated α-KG-dependent enzymes and bioenergetics pathways.

## MATERIALS AND METHODS

### Cell lines

Human U87MG glioblastoma, HEK-293TA, and HEK-293FT (ATCC; Manassas, VA) were maintained in DMEM medium (Mediatech; Herndon, VA) supplemented with 10% heat-inactivated fetal bovine serum (FBS) (Gemini Bio-Products; West Sacramento, CA), 2 mM L-glutamine (Life Technologies; Carlsbad, CA), and 100 U/mL penicillin and 100 μg/mL streptomycin (Genesee Scientific; San Diego, CA) herein referred as complete DMEM medium. Human U251 glioblastoma cell (ATCC) were maintained in complete DMEM medium supplemented with 1 mM sodium pyruvate, and non-essential amino acid cocktail (Genesee Scientific). Human umbilical vein endothelial cells (HUVEC) obtained from Fox Chase Cancer Center [FCCC] cell culture facility (Philadelphia, PA) were maintained in F-12K media supplemented with 10% FBS, 2 mM L-glutamine, 100 U/mL penicillin and 100 μg/mL streptomycin, 100 μg/mL heparin (Sigma-Aldrich), and EDGS cocktail (purified bovine serum albumin and bovine transferrin, hydrocortisone, rhIGF-1, PGE2, and rhEGF) (Life Technologies). Mouse lung endothelial cells (MLEC) generously donated by Dr. Jonathan Chernoff (FCCC) were cultured in EGM complete media kit (Lonza). TF-1 and TF-1a acute myeloid leukemia precursor cell lines, generously donated by Dr. Julie Losman (Harvard Medical School; Boston, MA) and Dr. Steven Sykes (FCCC), respectively, were both cultured in complete RPMI medium - only the cytokine-dependent TF-1 cells were cultured in 1 ng/mL rhIL-3. Human MLOM14 and U937 AML cell lines also provided by Dr. Steven Sykes and murine B16 melanoma cells (ATCC) were maintained in complete RPMI supplemented with 1 mM sodium pyruvate. HCT116 human colorectal cells were cultured in complete RPMI medium supplemented with 1 mM sodium pyruvate, non-essential amino acid cocktail, 10 mM HEPES, and 550 μM β-mercaptoethanol (Life Technologies). All cell lines were cultured at 37°C and 5% CO2.

Human cells were authenticated by IDEXX BioResearch (Columbia, MO) using STR profiling.

### Antibodies and chemical reagents

Annexin-V-FITC, FITC-anti-Rabbit IgG antibody, and PE-anti-Mouse IgG were purchased from Biolegend (San Diego, CA) and used for immunocytochemistry and flow cytometry analysis. Mouse anti-FLAG antibody (F1804) was obtained from Sigma. Anti-mouse secondary antibodies, AlexaFluor conjugated-far-red 647 (594/633 nm), and -yellow 532 (532/554 nm) were obtained from Invitrogen. Anti-β-actin antibody (60008–1) and anti-GAPDH-HRP (HRP-60004) were purchased from Proteintech (Chicago, IL). Antibody against IDH2 (ab55271) was purchased from Abcam (Cambridge, MA). Antibody against STAT3 (sc-482) was purchased from Santa Cruz Biotechnology, Inc. (Dallas, TX). Antibodies against phospho-STAT3-Y705 (9145S), FAK (3285-P) and phospho-FAK-Y397 (8556P) were purchased from Cell Signaling (Danvers, MA). Antibody against cMyc (LT0421) was purchased from Lifetein (South Plainfield, NJ) and anti-V5 antibody (600–401–378) from Rockland Immunochemicals (Pottstown, PA). Horseradish peroxidase (HRP)-conjugated goat anti-rabbit and anti-mouse antibodies were purchased from Invitrogen (Carlsbad, CA). Temozolomide, cisplatin, bortezomib, and vincristine were purchased from Millipore Sigma. Lyophilized (R)-2-HG and (S)-2-HG were generously provided by Dr. Ryan Looper (University of Utah). (R)-2-HG-TFMB was also synthesized by the Organic Synthesis Lab of FCCC.

### DNA vector constructs

Lentiviral plasmid pCDH-CMV-MCS-GFP-EF1-puro (System Biosciences; Mountain View, CA), was used to subclone wild type IDH2 cDNA from the pLenti-IDH2 vector (provided by Dr. Hai Yan, Duke University). FLAG sequence was added to the 3′ end of IDH2 before a stop codon by standard PCR using the following primers: Forward- 5′-CGACTCGAGCAGATGGCCGGCTACCTG-3′ and Reverse-3-GGTGGATCCTTACTACTTATCGTCGTCATCCTTGTAATCCTGCCTGCCCAGGGC-5′ (Bioneer). Site-directed mutagenesis was next performed using QuikChange Lightning kit (Agilent Technologies, Santa Clara, CA) to construct R172K, R172M, and R140Q-IDH2 mutants.

### Lentiviral and retroviral transduction

N-RAS and empty vector control plasmid (pBabe) retroviruses were generously supplied by Dr. Steven Sykes (FCCC). IDH2 lentivirus was produced using the lentivirus expression packaging system (Invitrogen) and 293FT cells following the manufacturer’s protocol. Supernatant containing lentivirus was collected 72 hours later and passed through a 0.45 μM filter. Cells were transduced with lentivirus or retrovirus in the presence of 1 μg/mL polybrene (Sigma) and subjected to antibiotic selection by adding 5 μg/mL puromycin (Life Technologies) to the media three days later post-infection. Pooled populations were then maintained in 3 μg/mL puromycin.

### Western blot and immunoprecipitation

Cells were lysed in TritonX-100 lysis buffer (50 mM Tris pH 7.5, 150 mM NaCl, 2 mM EDTA, 0.5% TritonX-100) supplemented with 1X Protease Inhibitor Cocktail (Roche Diagnostics), 1 mM sodium orthovanadate, 1 mM PMSF and 1X phosphatase inhibitor mixture 1 and 2 (Sigma), for 20 minutes on ice. After centrifugation for 15 minutes at 4°C supernatants were collected and protein concentration determined by Bio-Rad protein assay kit (Bio-Rad). Immunoprecipitation assays were performed by first pre-clearing protein lysates by incubation with Protein G-Sepharose (GE Healthcare; Mickleton, NJ). Pre-cleared lysates were then incubated with the indicated antibody overnight at 4°C and immunocomplexes collected by further addition of Protein G-Sepharose for 2 hours. Lysates and immunoprecipitates were resolved on NuPAGE 4–12% Bis-Tris gels (GenScript USA Inc, Piscataway, NJ) and transferred to PVDF membranes (Millipore Sigma). Membranes were blocked with Blocker Casein TBS (Thermo Scientific, Rockford, IL) and incubated with the corresponding primary and HRP-conjugated secondary antibodies in TBS-T + 3% BSA. Membranes were developed by chemiluminescence using SuperSignal West Pico or Femto (Thermo Scientific). Images were captured with Alpha-Innotech HD2 imaging system (ProteinSimple, CA) and quantified using AlphaView software.

### Proliferation assays

Cell proliferation was determined by manual cell counts using a hemacytometer. Cells were seeded in triplicate in a 12-well plate at a density of 2 × 10^5^ and counted at days 3 and 5. For long-term cell count experiments (e.g. 28 or 50 days), cell counts were done by serial re-plating of 10,000 cells in a 60 mm plate every 7 days. Fold change was calculated by compounding all fold change counts from each experiment based on original and following cell counts. Changes in proliferation were also determined by using CellTiter 96^®^ AQueous One solution reagent (Promega, Madison, WI) according to the manufacturer’s protocol. Cells were seeded in flat-bottomed 96-well plates at a density of 500 cells per well and treated with or without the indicated drug and days at 37°C. Absorbance (Abs) was measured at 490 nm after a 2-hour incubation using a VICTOR^™^*X*5 Multilabel Plate Reader (PerkinElmer Life Sciences; Waltham, MA). Background values were first subtracted from each well before proceeding with data analysis. Percent growth inhibition from control was determined with the following formula = 1 – (Abs-treated/Abs-control) × 100.

### Soft-agar colony formation assay

Experiments were performed in 6-well plates. The base layer was comprised of 2 mL of DMEM with 0.6% bacto-agar (Becton-Dickinson; Franklin Lakes, NJ) and allowed to solidify. Cells (2 × 10^4^ cells per well) were resuspended in 2 mL of DMEM with 0.6% bacto-agar and overlaid onto the base layer. Plates were maintained at 37°C for 28 days. Two hundred μL of complete DMEM was added to each well weekly to prevent agar from drying. Colonies were photographed at 1X magnification and colonies counted by using the colony count program within the AlphaView software.

### Wound healing assay

Cells were seeded the day before in triplicate wells in a 12-well plate at a density of 1 × 10^6^ per well. Cells were then pre-treated with 3 μg/mL mitomycin-c (Sigma) to stop proliferation and discern differences in migration, followed by labeling with 1 μM calcein-AM tracking dye (Life Technologies). A single straight-line wound was then made through the middle of each well using a 200 μL pipet tip. The wells were washed with 1X PBS to remove floating cells. Images of individual wells were taken immediately and after 18 hours using a Nikon Eclipse TE-2000 U fluorescent microscope. Analysis of the wound area was then performed with the colony count program of the AlphaView software.

### Cell invasion assay

Cell invasion was determined using the 24-well Transwell Matrigel Invasion kit purchased from Corning (Tewksbury, MA). Twenty-five thousand cells were seeded in each invasion chamber and 10% FBS was used as chemoattractant. After 24 hours, the invasion membranes were fixed and stained using the 3-stain Set (Thermo-Fischer Scientific; Waltham, MA). Images of processed membranes were taken at 4X magnification and cells counted to quantitate the degree of invasive capability.

### Apoptosis assay

One million cells were collected and washed in 3 mL of PBS. Cells were then dually stained with FITC Annexin-V (Biolegend) for 20 minutes in 1X binding buffer (10 mM HEPES, 140 mM NaCl, 2.5 mM CaCl_2_, pH 7.4) at room temperature in the dark. Ten thousand cells were collected using BD FACS Calibur Flow Cytometer (BD; Franklin Lakes, NJ) and data analyzed using FlowJo software (Ashland, OR).

### (R)-2-HG measurement

Approximately 4–8 million cells per sample (~100 uL pellet volume) were centrifuged, washed twice with 1X PBS and snap-frozen in dry ice. Cell pellets were analyzed by gas chromatography followed by mass spectroscopy (GC-MS) at the Frederick National Laboratory for Cancer Research (Frederick, MD). Concentrations of (R)-2-HG are reported as the amount in weight by amount of total cell pellet protein (w/w).

### NADPH/NADP^+^ ratio measurement

Cells were disrupted in lysis buffer (50 mM Tris pH 7.5, 150 mM NaCl, 2 mM EDTA pH 8.0, 0.5% TritonX-100, complete Mini protease inhibitors [Roche; Indianapolis, IN], 100 μM phenylmethylsulfonyl fluoride (PSMF), and 100 μM sodium orthovanadate). Cell debris was removed by centrifugation at 12,000 × g and 4°C for 15 minutes. The supernatant fraction was collected, and protein concentration was determined at 590 nm using Bio-Rad Bradford Protein Assay (Biorad). NADP/NADPH fluorometric assay kit (Abcam) was used as directed by the manufacturer to determine intracellular ratios of these metabolites. 590 nm absorbance was read with a VictorX5 96-well plate reader (Perkin-Elmer).

### ATP measurement

Cells at a density of 3 × 10^6^ per sample were washed twice in PBS and lysed in 300 μL of double distilled, osmotically filtered water for 10 minutes on ice. Lysates (100 μL) in triplicate were then transferred to a 96-well flat-bottomed plate. CellTiter Glo Luminescent reagent (Promega) was used following the manufacturer’s protocol. Luminescence was taken at one- second exposure with a VictorX5 96-well plate reader.

### Immunocytochemistry

Cells were plated on poly-L-lysine coated slides (Sigma-Aldrich; St. Louis, MO), fixed in 4% paraformaldehyde for 1 hour and permeabilized with 0.1% TritonX-100 for 5 minutes. Slides were then blocked in 4% goat serum, incubated with anti-IDH2 antibody followed by incubation with FITC-anti-mouse IgG secondary antibody. Nuclei were counterstained with VectaShield DAPI mounting media (VectorLabs; Burlingame, CA). Images were acquired using Leica Sp5 Confocal Microscope (Buffalo Grove, IL). To label mitochondria, cells were pre-treated with 100 nM Mitotracker Red CMX Ros (Invitrogen) for 1 hour before washing with PBS and fixation.

### Animals

*Rag1*^−/−^ and SCID mice were purchased from The Jackson Laboratory (Bar Harbor, ME). C57/BL6 wild type mice were purchased from the Animal Production Area of the Frederick National Laboratory for Cancer Research (Frederick, MD). Mice were bred in our animal facility and housed in a pathogen-free environment. Animal studies were performed in accordance with the National Institute of Health Guide for Care and Use of Laboratory Animals and approved by Temple University Animal Care and Use Committee guidelines.

### Tumor transplantation

U87MG (5 × 10^6^), B16-F1 (1 × 10^6^), or HCT116 (5 × 10^6^) tumor cells in 200 μL of endotoxin-free saline solution were injected subcutaneously on the dorsal flank of 6- to 8-weeks old *Rag1*^−/−^ or SCID mice. Tumor measurements were started at day 7 using a digital caliper (Neiko Tools; Japan). Tumor volume was determined with the formula: *V = a*^2^*b*, where *a* is the shorter diameter and *b* is the longer diameter of the tumor.

### Orthotopic intracranial tumor xenografts

Eight 10-week-old *Rag1*^−/−^ female mice were used to implant glioblastoma tumor cells in the brain. Animals were prepared as outlined in [57]. A 0.75 cm incision was made longitudinally in the mid-scalp extending from the level of the eyes caudally and a small hole was drilled in the skull using the following coordinates: 2 mm in front of bregma and 2 mm to the left of center on top of the head. Tumor cells (1.5 × 10^4^) in 3 μL volume were injected 3 mm intracranially with a Hamilton syringe (Hamilton, Reno, NV). The scalp and hole in skull were sealed with VetBond bonding agent (3M; St. Paul, MN). Animals were monitored daily and at the end of the study animals were processed and analyzed as described in [57]. Brain volume was determined by calculating the area of tumor in each section and multiplying by the thickness of tissue (5 μM). Images were acquired using NIS Elements imaging software (Nikon) and subsequently analyzed for glioblastoma tumor volume using ImageJ software (NIH).

### IDH2 structural modeling

IDH2 homology modeling was used to construct structural models of both wild type and mutant IDH2 in the different states of interest. Models of human IDH2 were built using as templates the solved crystal structures of human IDH1 (PDB codes 1T09, 1T0L, 3MAR, 3MAP, 3INM and 4KZ0 [27, 28, 32, 33, 58]) and yeast IDH2 (PDB code 2QFX [59]). The multiple sequence alignment between human IDH2 and the templates is shown in Supplementary Figure 1. Modeler (version 9.10) [60, 61] was employed for pairwise structure-sequence alignment and homology model building and representative models were selected using the g_cluster tool of the Gromacs package [62]. The crystal structures of the R132H IDH1 mutant used as templates of the open and quasi-open states of the neomorphic reaction (PDB codes 3MAR and 3MAP [27] have missing segments (residues 135–140 and 272–285 for 3MAR and residues 135–139 and 273–280 for 3MAP), which were added using the loop modeling application in Rosetta (version 3.2.1) [63]. Structural images were generated with VMD [64] and visualization of the multiple sequence alignment was carried out with ESPript [65].

### Statistical analysis

Prism software (GraphPad, San Diego) was used for statistical analysis. Results were analyzed using the Student’s *t*- test to assess the significance and two-tailed ANOVA. Values of ^*^p ≤ 0.05, ^**^p ≤ 0.01, and ^***^p ≤ 0.001 were considered statistically significant. Error bars were shown to represent standard error of the mean (SEM).

## SUPPLEMENTARY MATERIALS TABLES AND FIGURES



## Abbreviations

(R)-2-HG: (R)-2-hydroxyglutarate [(D)-2-hydroxyglutarate]
(R)-2-HG-TFMB: (R)-2-hydroxyglutarate-3-trifluoromethylbenzyl
(S)-2-HG: (S)-2-hydroxyglutarate [(L)-2-hydroxyglutarate]
HIF-1α: hypoxia-inducible factor 1-alpha
IDH1: Isocitrate Dehydrogenase-1
IDH2: Isocitrate Dehydrogenase-2
IDH3: Isocitrate Dehydrogenase-3
K_cat_: catalytic constant
K_m_: Michaelis constant
L2HGDH: (L)-2-hydroxyglutarate dehydrogenase [(S)-2-hydroxyglutarate dehydrogenase]
MDH: malate dehydrogenase
TET1: Ten-eleven translocation methylcytosine dioxygenase-1
TET2: Ten-eleven translocation methylcytosine dioxygenase-2
TMZ: temozolomide
α-KG: alpha-ketoglutarate
