# Supplementary material for: Characterization of cancer-associated IDH2 mutations that differ in tumorigenicity, chemosensitivity and 2-hydroxyglutarate production
Source: Oncotarget. 2019 Apr 12;10(28):2675–92. doi: 10.18632/oncotarget.26848 (PMC6505628; doi:10.18632/oncotarget.26848)
Supplement: Supplementary file 1 [file oncotarget-10-2675-s001.pdf]

Characterization of cancer-associated IDH2 mutations that differ in tumorigenicity, chemosensitivity and 2-hydroxyglutarate production

SUPPLEMENTARY MATERIALS

REFERENCES

1. Xu X, Zhao J, Xu Z, Peng B, Huang Q, Arnold E, Ding J. Structures of human cytosolic NADP-dependent isocitrate dehydrogenase reveal a novel self-regulatory mechanism of activity. J Biol Chem. 2004; 279:33946–57. https://doi.org/10.1074/jbc.M404298200.
2. Yang B, Zhong C, Peng Y, Lai Z, Ding J. Molecular mechanisms of "off-on switch" of activities of human IDH1 by tumor-associated mutation R132H. Cell Res. 2010; 20:1188–200. https://doi.org/10.1038/cr.2010.145.
3. Dang L, White DW, Gross S, Bennett BD, Bittinger MA, Driggers EM, Fantin VR, Jang HG, Jin S, Keenan MC, Marks KM, Prins RM, Ward PS, et al. Cancer-associated IDH1 mutations produce 2-hydroxyglutarate. Nature. 2009; 462:739–44. https://doi.org/10.1038/nature08617.
4. Rendina AR, Pietrak B, Smallwood A, Zhao H, Qi H, Quinn C, Adams ND, Concha N, Duraiswami C, Thrall SH, Sweitzer S, Schwartz B. Mutant IDH1 enhances the production of 2-hydroxyglutarate due to its kinetic mechanism. Biochemistry. 2013; 52:4563–77. https://doi.org/10.1021/bi400514k.
5. Peng Y, Zhong C, Huang W, Ding J. Structural studies of Saccharomyces cerevesiae mitochondrial NADP-dependent isocitrate dehydrogenase in different enzymatic states reveal substantial conformational changes during the catalytic reaction. Protein Sci. 2008; 17:1542–54. https://doi.org/10.1110/ps.035675.108.
6. Wang F, Travins J, DeLaBarre B, Penard-Lacronique V, Schalm S, Hansen E, Straley K, Kernytsky A, Liu W, Gliser C, Yang H, Gross S, Artin E, et al. Targeted inhibition of mutant IDH2 in leukemia cells induces cellular differentiation. Science. 2013; 340:622–26. https://doi.org/10.1126/science.1234769.

Supplementary Table 1: Summary of effects to the catalytic site with 3 IDH2 mutants

**Wild-type reaction**			
	**R172K**	**R172M**	**R140Q**
Open state	altered open-to-closed transition	altered open-to-closed transition	-
Quasi-open state	-	-	reduced ICT binding at the secondary site
Closed state	lowered affinity for ICT at the catalytic site	lowered affinity for ICT at the catalytic site	lowered affinity for ICT at the catalytic site
**Neomorphic reaction**			
	**R172K**	**R172M**	**R140Q**
Open state	-	-	-
Quasi-open state	-	-	-
Closed state	unaltered α-KG binding, but only partially hindered Y179 rotation	reduced α-KG / ICT competition and favorable Y179 rotation	reduced α-KG / ICT competition, but prohibited Y179 rotation

Proposed effects of the IDH2 mutations in the different enzymatic states relevant for the normal and neomorphic reaction.

Supplementary Table 2: Structural templates used in the homology modeling of IDH2 in the different enzymatic states relevant for the normal and neomorphic reactions

Normal reaction		
	**WT**	**Mutant**
Open state (IDH2–NADP$^+$)	1T09	*virtual* mutation on 1T09, 4JA8
Quasi-open state (IDH2–NADP$^+$–ICT $^{site\ II}$)	*virtual* back-mutation on 3MAP	3MAP
Closed state (IDH2–NADP$^+$–ICT $^{site\ I}$)	1T0L	*virtual* mutation on 1T0L
**Mutant reaction**		
	**WT**	**Mutant**
Open state (IDH2–NADPH)	*virtual* back-mutation on 3MAR	3MAR
Quasi-open state (IDH2–NADPH–α-Kg $^{site\ II}$)	*virtual* back-mutation on 3MAP	3MAP
Closed state (IDH2–NADPH–α-KG $^{site\ I}$)	2QFX	3INM, 4KZ0

The PDB codes 1T09 and 1T0L [1] correspond to human WT-IDH1, 3MAR, 3MAP [2], 3INM [3] and 4KZ0 [4] to human R132H-IDH1, 2QFX [5] to yeast WT-IDH2 and 4JA8 [6] to human R140Q-IDH2.

Supplementary Table 3: Summary of phenotypes from glioblastoma cells expressing three clinically relevant IDH2 mutations

**All relative to IDH2-WT, U87MG+IDH2**	**R172K**	**R172M**	**R140Q**
Proliferation	No change	↓	↑
Migration	No change	↓	↑
Invasion	↑	↓↓	↑↑
Chemosensitivity (TMZ)	↑	↓	↓
Tumor colony formation	No change	↓	↑
Tumorigenicity, xenograft	No change	↓↓	↑↑
(R)-2-HG production	↑↑	↑↑↑	↑

All relative to IDH2-WT expressing cells.

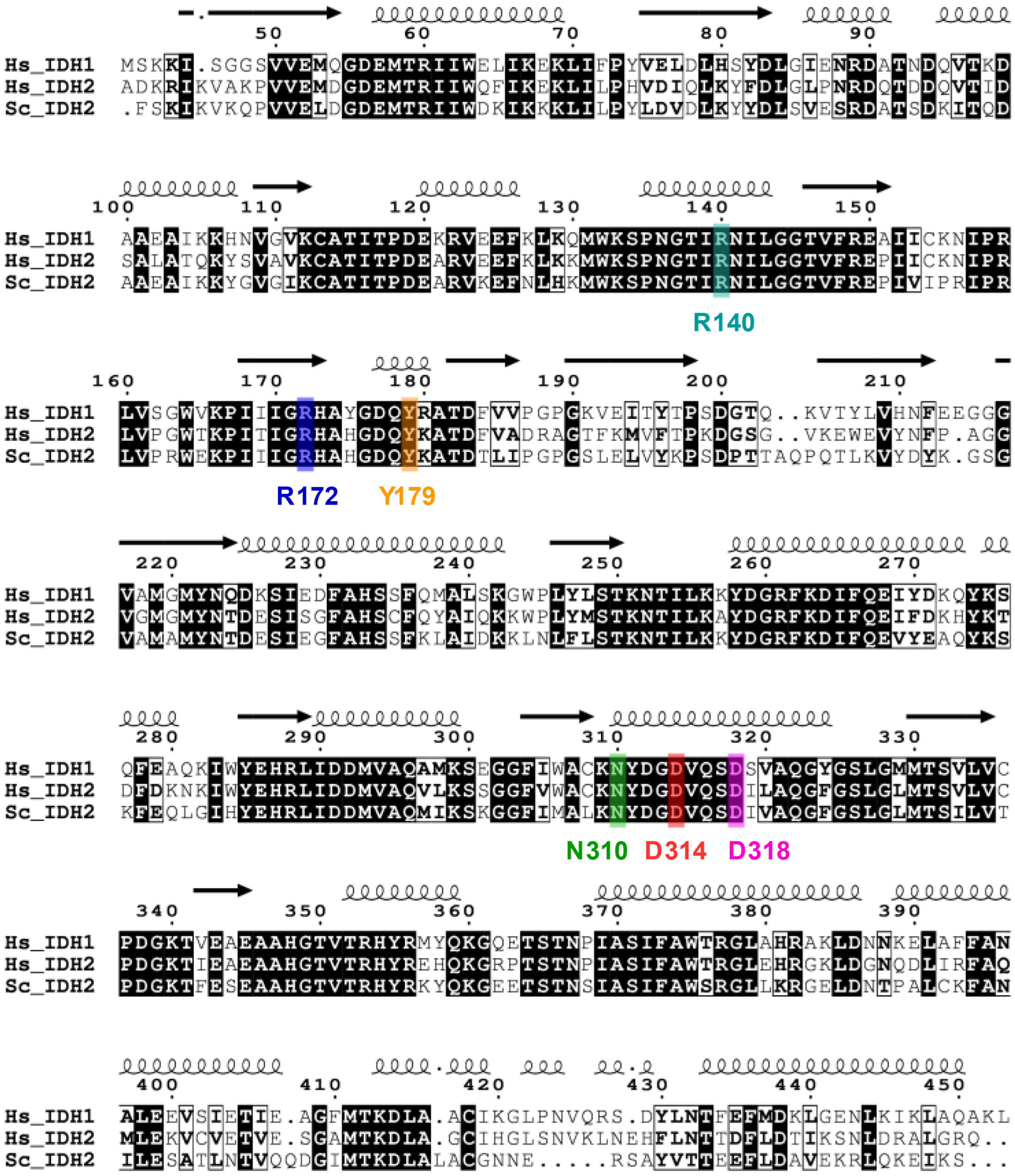

Supplementary Figure 1: Multiple sequence alignments of the IDH2 and IDH1. Sequence alignment of human IDH2 (Hs_IDH2) and the two homologs used as templates in the structural modeling, human IDH1 (Hs_IDH1) and yeast IDH2 (Sc_IDH2). The residue numbering of human IDH2 is shown above the aligned sequences, together with the secondary structure elements predicted for the human IDH1 structure (PDB code 1T0L). Conserved residues are highlighted in black, and similar residues are boxed. Human IDH2 residues discussed in the text are indicated with colors.

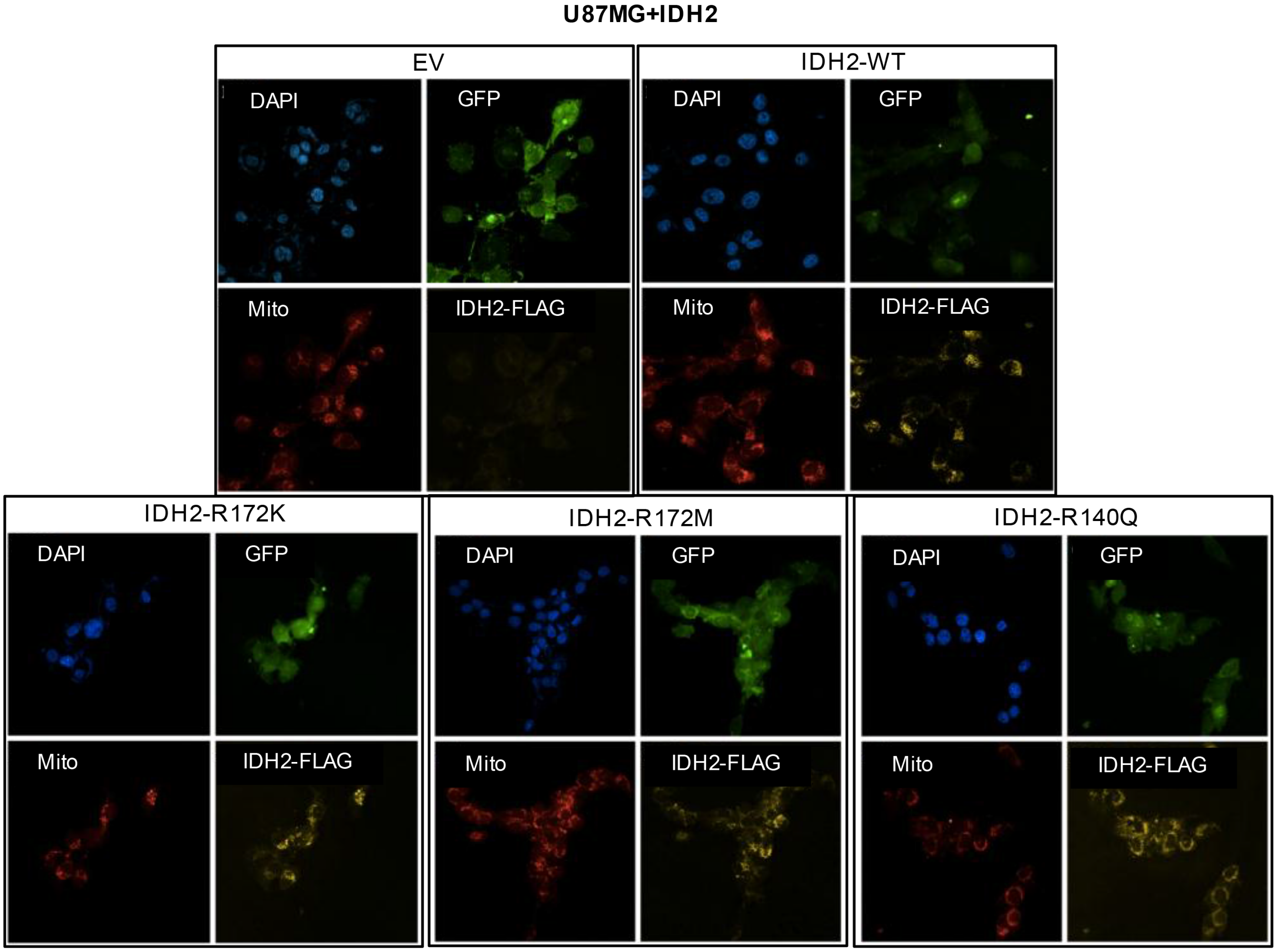

Supplementary Figure 2: Mitochondrial localization of U87MG-IDH2 variants. Confocal images were taken of U87MG cells transduced with empty lentiviral vector (EV), lentivirus encoding wild type (WT) or individual IDH2 mutants (R172K, R172M, and R140Q). Cells were pre-treated with Mitotracker (Mito; mitochondria, red) followed by anti-FLAG immunostaining (IDH2, yellow) and counter-stained with DAPI (nucleus, blue). The lentiviral vectors carry the GFP marker (green), which was used as a positive control for efficient transduction. One representative image is shown of each U87MG cell variant at 20X magnification.

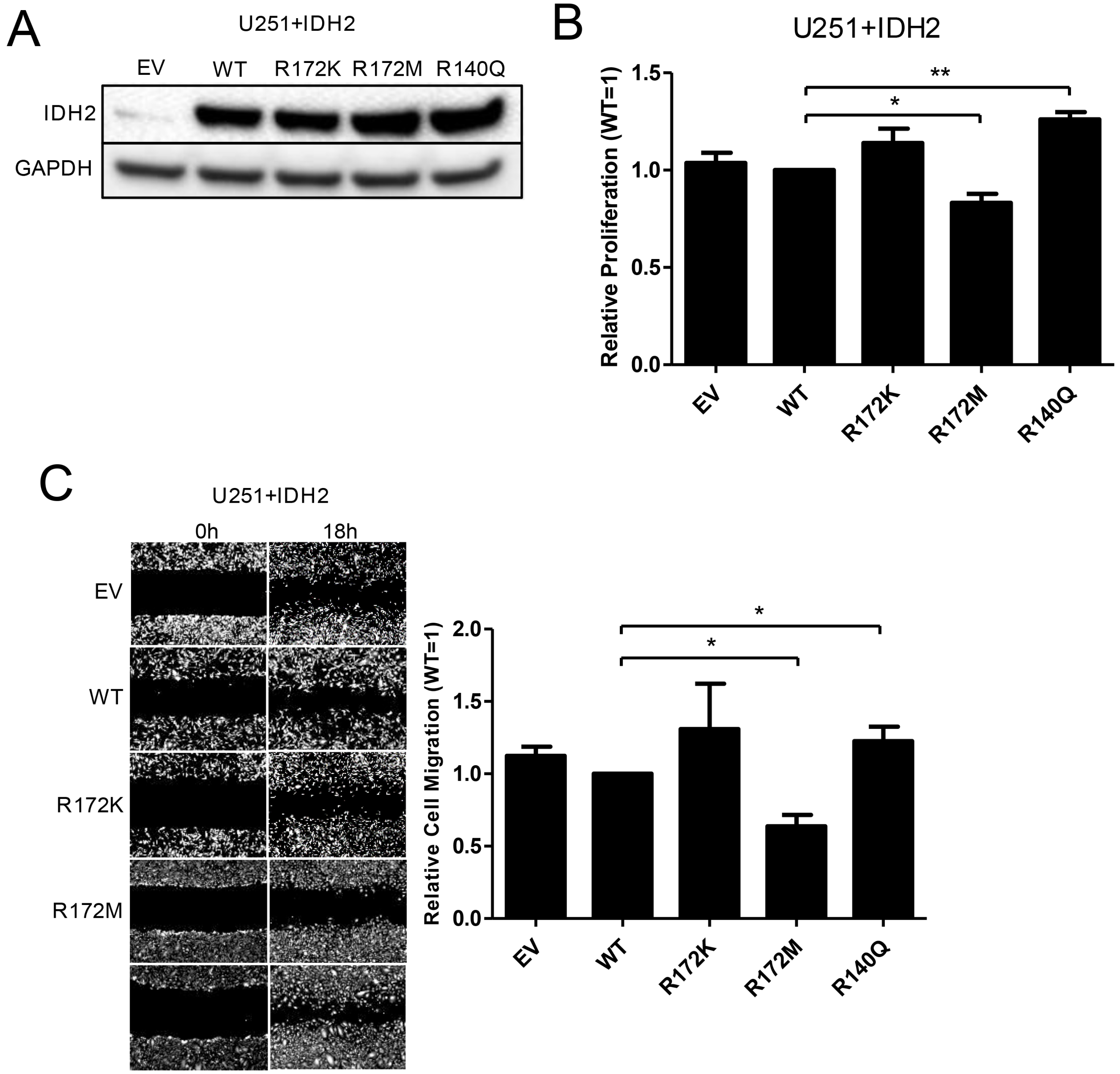

Supplementary Figure 3: IDH2 mutations alter the tumorigenic properties of U251 glioblastoma cells. Overexpression of either wild type (WT) or individual clinically relevant IDH2 mutations (R172K, R172M, and R140Q) in U251 cells was confirmed by Western blot analysis (**A**). Cell proliferation was determined by manual cell counts, over 96 h and normalized to WT values (**B**). Assessment of cell migration via wound healing assay by comparing differences in cell density after 18 h, relative to WT values (**C**). Shown are representative images taken at 4X magnification from three independent experiments. $^{*}p \leq 0.05$.

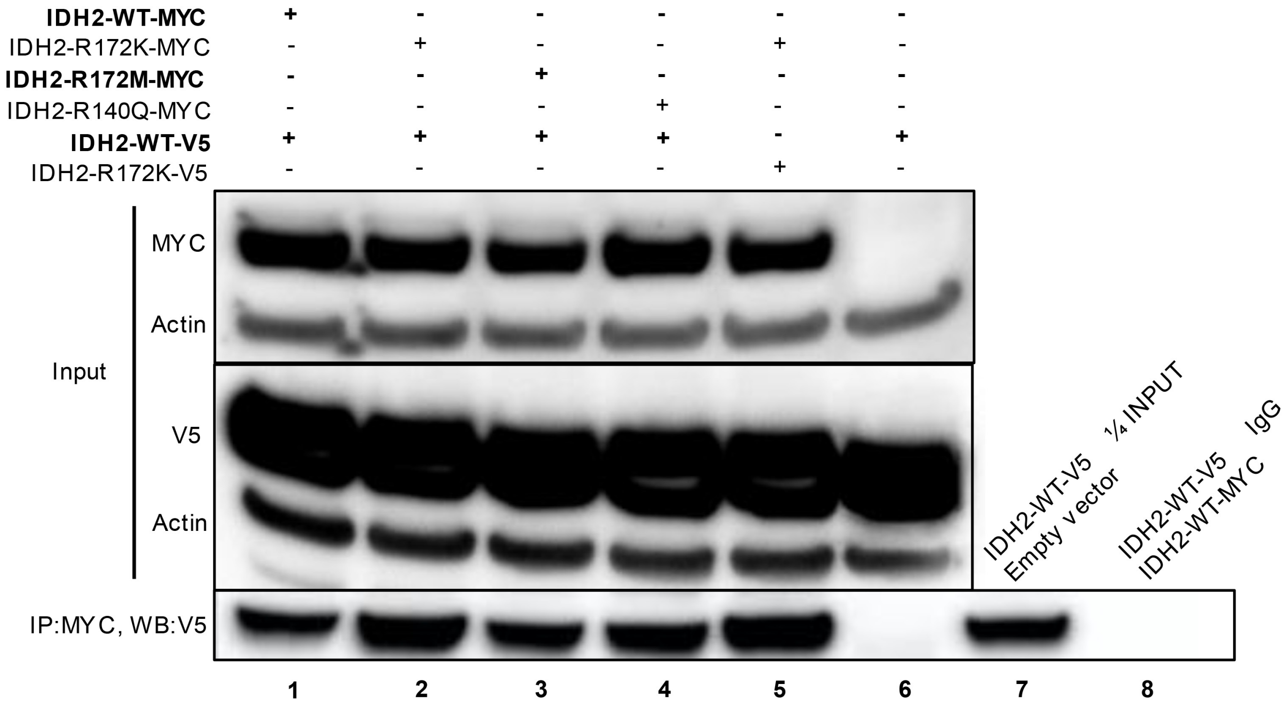

Supplementary Figure 4: IDH2 dimerizes with mutant IDH2 irrespective of the nature of the IDH2 mutation. HEK-293TA cells were transiently co-transfected with V5-tagged IDH2-WT and MYC-tagged IDH2 (IDH2-WT, IDH2- R172K, IDH2-R172M, or IDH2-R140Q). V5-immunoprecipitates were evaluated for interactions with MYC-tagged IDH2-WT or mutant IDH2 by Western blot analysis. Non-specific immunoglobin (IgG) was used as a negative control for non-specific binding. Shown is a representative experiment of three that were performed.

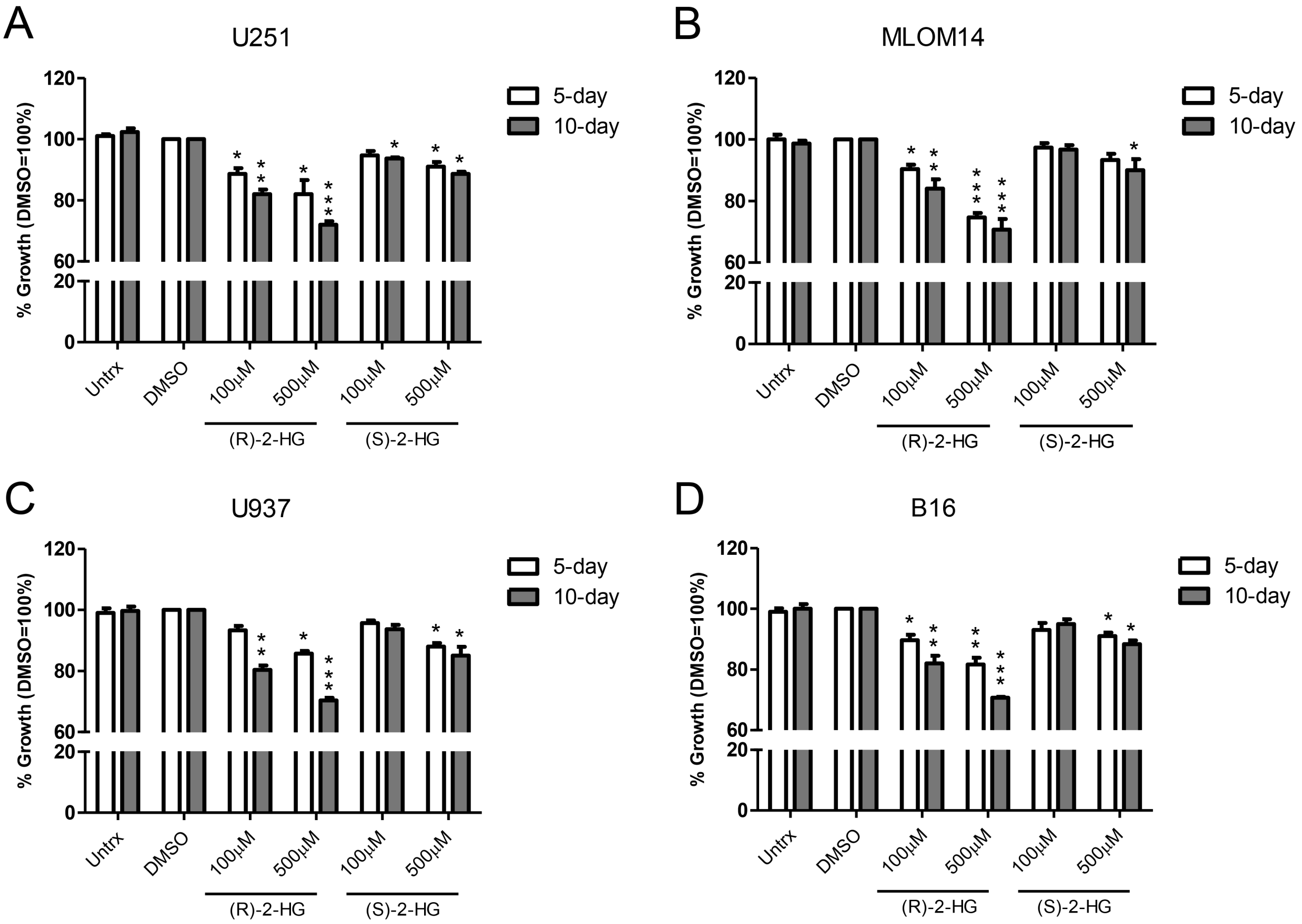

Supplementary Figure 5: (R)-2-HG inhibits the proliferation of several tumor cell lines. U251, MLOM14, U937 and B16 tumor cells which carry wild type IDH2 were treated with vehicle control (DMSO), 100 µM or 500 µM of exogenous (R)-2-HG or (S)-2-HG for 5 and 10 days. Total cell number was determined and normalized to vehicle control (**A**–**D**). Data are shown as mean ± SEM of three independent experiments. $^{*}p \leq 0.05$; $^{**}p \leq 0.01$; $^{***}p \leq 0.001$.
